# Coformulation of Broadly Neutralizing Antibodies 3BNC117 and PGT121: Analytical Challenges During Preformulation Characterization and Storage Stability Studies

**DOI:** 10.1016/j.xphs.2018.08.012

**Published:** 2018-12

**Authors:** Ashaben Patel, Vineet Gupta, John Hickey, Nancy S. Nightlinger, Richard S. Rogers, Christine Siska, Sangeeta B. Joshi, Michael S. Seaman, David B. Volkin, Bruce A. Kerwin

**Affiliations:** 1 Department of Pharmaceutical Chemistry, Macromolecule and Vaccine Stabilization Center, University of Kansas, 2030 Becker Drive, Lawrence, Kansas 66047; 2 Just Biotherapeutics Inc., 401 Terry Avenue North, Seattle, Washington 98109; 3 Beth Israel Deaconess Medical Center, Boston, Massachusetts 02215

**Keywords:** broadly neutralizing antibodies, stability, coformulation, mass spectrometry, protein aggregation, calorimetry (DSC), capillary electrophoresis, deamidation, oxidation, biotechnology

## Abstract

In this study, we investigated analytical challenges associated with the formulation of 2 anti-HIV broadly neutralizing antibodies (bnAbs), 3BNC117 and PGT121, both separately at 100 mg/mL and together at 50 mg/mL each. The bnAb formulations were characterized for relative solubility and conformational stability followed by accelerated and real-time stability studies. Although the bnAbs were stable during 4°C storage, incubation at 40°C differentiated their stability profiles. Specific concentration-dependent aggregation rates at 30°C and 40°C were measured by size exclusion chromatography for the individual bnAbs with the mixture showing intermediate behavior. Interestingly, although the relative ratio of the 2 bnAbs remained constant at 4°C, the ratio of 3BNC117 to PGT121 increased in the dimer that formed during storage at 40°C. A mass spectrometry-based multiattribute method, identified and quantified differences in modifications of the Fab regions for each bnAb within the mixture including clipping, oxidation, deamidation, and isomerization sites. Each bnAb showed slight differences in the levels and sites of lysine residue glycations. Together, these data demonstrate the ability to differentiate degradation products from individual antibodies within the bnAb mixture, and that degradation rates are influenced not only by the individual bnAb concentrations but also by the mixture concentration.

## Introduction

The increasing use of passive immunization strategies to treat unmet medical needs is delineating the need for coformulation and codelivery of defined monoclonal antibody mixtures. Monoclonal antibodies (Abs) are increasingly being developed for passive immunization strategies against multiple types of pathogens. This is especially important due to the emergence of antibiotic-resistant bacteria,^1^ long development timelines for prophylactic vaccines,^2^ and the fragile nature of vaccine supply.^2^, ^3^, ^4^ Abs in development include those against organisms targeted for biodefense such as anthrax, plague, and botulinum toxin,^5^ the influenza surface glycoprotein hemagglutinin,^6^ Abs against each of the virulence components of anthrax,^5^, ^7^ mAbs against ebola virus,^8^, ^9^, ^10^, ^11^, ^12^ filovirus,^8^ rabies,^13^, ^14^ and human immunodeficiency virus (HIV).^15^, ^16^, ^17^, ^18^, ^19^, ^20^, ^21^ Associated with these pathogens is a high degree of genetic variability such as that observed with the viral clades of the H5N1 virus^22^, ^23^ and the multiple viral clades of HIV.^24^ This high degree of genetic diversity, and in turn diversity in the structure of highly immunogenic surface-exposed antigens such as the influenza glycoprotein hemagglutinin and the gp120 protein of HIV is combatted by our immune systems with development of Abs with broad neutralization abilities against multiple viral subtypes, termed broadly neutralizing Abs (bnAbs).^25^, ^26^ In contrast to bnAbs, many of the monoclonal Abs developed for diseases such as inflammation, heart disease, and cancer are highly specific for single epitope without the mutational diversity observed for pathogens.^27^

One promising approach for coformulation and codelivery of mAbs for passive immunization is the use of multiple broadly neutralizing anti-HIV monoclonal Abs (bnAbs), each targeting different structural aspects of the gp120 coat protein. Animal studies and clinical trials of bnAbs have demonstrated the need for coinfusion of Abs against multiple targets of the immunogen to prevent viral escape.^16^, ^28^ For example, gp120 surface glycoprotein of HIV is highly immunogenic at the CD4 binding site, across multiple sites of the glycan shield, and the membrane proximal external region.^29^ Viral escape occurs due to its continuing mutational diversity. In some individuals though, disease has been held in check through the maturation of multiple Abs that can neutralize virus with a broad array of mutations at individual epitopes. These include the bnAb 3BNC117 against the CD4 binding region of gp120, the bnAbs 10-1074, and PGT121 against the V1/V2 glycan and the bnAb PGDM1400 against the trimer apex of the HIV envelope. Because these bnAbs cannot individually neutralize all genetic variants of HIV, combinations of 2 or more bnAbs against different epitopes have been suggested for neutralization of up to 99% of viral clades.^16^

Today, approximately 75 monoclonal Abs have been commercialized as biotherapeutics against a wide array of diseases.^30^ The mAbs have been developed as single mAb entities with a typical drug product (DP) containing a narrow range of excipients generally including a buffer, polyol or amino acid, a surfactant, and possibly salts.^31^, ^32^, ^33^ Characterization of the DP includes assays for potency, aggregation, fragmentation, and posttranslational modifications (PTMs) such as oxidation, deamidation, isomerization, and glycation. Typical assays include size exclusion chromatography,^34^, ^35^ visible and subvisible particle analysis,^36^, ^37^ capillary electrophoresis (CE)^38^ for clipping and ion-exchange chromatography^39^ or capillary isoelectric focusing^38^ for PTMs. These chromatographic and electrophoretic methods, although classically used to monitor product quality during stability studies of biologics are not able to monitor changes at the molecular level. For example, while clipping may be observed, the site of clipping cannot be assessed by CE. In corollary, changes in the overall charge can be assessed by ion-exchange chromatography, but the type and site of modification cannot be assessed by the technique. To obtain this information, successive steps of enzymatic digestion, followed by chromatographic separation and mass spectrometric analysis have been used to define modifications such as deamidation and oxidation, and the site of degradation within the protein. Because of analytical and instrumental complexities, this peptide mapping technique has been used primarily for characterization purposes and has only found limited use during routine DP stability studies.

Application of these commonly used analytical techniques to coformulated Abs is more difficult to interpret. Coformulation of Abs has only been carried out for a limited number of products focused on polyclonal Abs and short term formulation studies of monoclonal antibody-based products. Products such as intravenous immunoglobulin^40^ are polyclonal in nature and multiple publication have described aggregation,^41^ fragmentation,^42^ and oxidation of the Abs in mixtures.^43^ It is difficult to define the degradation beyond the effect on classes of IgG, that is, idiotype/anti-idiotype aggregate complexes, and how degradation of specific IgG’s affects function is not possible. Degradation in known mixtures of Abs is easier to interpret but has only been done in a limited number of cases^44^, ^45^, ^46^, ^47^ and only for short term stability studies. In a series of studies using cation-exchange chromatography to separate individual mAbs in a mixture, the effects of various stresses, pH, salt, temperature, and vortexing were investigated. Precipitation of the individual mAbs in the mixtures occurred by self-association, with differing stabilities observed for each of the mAbs.^44^ Glover et al. investigated the coformulation of Pertuzumab and Trastuzumab, 2 IgG1 mAbs, in an intravenous infusion bag containing 0.9% sodium chloride and found similar stability trends for the mixture versus the same mAbs alone following incubation at 30°C for 24 h.^47^ Similar to the intravenous immunoglobulin products, the majority of the analytical techniques, except for ion-exchange chromatography, did not distinguish between the mAb species within the mixture.

In the studies described here, our 2 main goals were (1) to demonstrate that coformulated mAbs could be formulated and stored under conditions commonly used for commercial mAbs during long-term storage; and (2) to demonstrate the utility of the multiattribute method (MAM)^48^ for characterizing degradation products in singly and coformulated mAb mixtures. The first goal is important for developing platform formulations for use in early clinical trials allowing for faster movement to the clinic and reduction in development costs. The second goal drives us toward a molecular level characterization of mAb stability during storage, an area that has been lacking for mixture studies. Toward our first goal, we first examined the relative solubility profile (using a polyethylene glycol [PEG] precipitation assay) and conformational stability (using differential scanning calorimetry [DSC]) to establish some baseline physical properties of the bnAb formulations. Using accelerated and real-time stability studies, we then show that coformulated mAbs are stable in an acetate/sucrose/polysorbate formulation at 100 mg/mL, and that the degradation of the mAbs in the mixture approximated that seen for the individually formulated mAbs. Along with the more standard analytical tools such as size exclusion high-performance liquid chromatography (SE-HPLC), microflow imaging, dynamic light scattering, and CE-sodium dodecyl sulfate (CE-SDS), we used the mass spectrometry-based MAM to gain molecular level characterization of degradants, identifying deamidation, isomerization, glycation, and oxidation sites specific to each mAb in the mixture within the Fv region. Because both molecules were IgG1, we were not able to differentiate when modifications, in the constant domains, were specific to either molecule. An added difficulty to these mixtures is characterizing components of the aggregates. Toward that end, we used the MAM technique to quantify the relative levels of each bnAb in the mixture and identify PTMs associated with the aggregated bnAbs. Together, these data demonstrate that complex mixtures of mAbs can be characterized at the molecular level providing greater insight into stability of specific regions of the proteins during storage.

## Materials and Methods

### Materials

The 3BNC117 bnAb was the kind gift of Dr. Michel Nussenzweig (Rockefeller University, New York, NY) and the PGT121 bnAb was the kind gift of Dr. Dan Barouch (Harvard University, Boston, MA). Frozen stocks of 3BNC117 (extinction coefficient 1.69 [(mg/mL)^-1^ cm^-1^]) and PGT121 (extinction coefficient 1.72 [(mg/mL)^-1^ cm^-1^]) at ∼100 mg/mL, each were prepared at Just Biotherapeutics (Seattle, WA) and sent to the University of Kansas for vialing and stored at −80°C. A mixture of 3BNC117 and PGT121 (extinction coefficient for mixture 1.69 [(mg/mL)^-1^ cm^-1^]) was prepared by mixing the 2 stock bnAbs, each formulated in 20 mM acetate, 9% sucrose, 0.01% w/v polysorbate 80 (PS80) pH 5.2 (ASu), at a 1:1 volume ratio. The details of the composition of each bnAb formulation are listed in Table 1. Most chemicals and reagents were purchased from Fisher Scientific (Fair Lawn, NJ) and Sigma Aldrich (St. Louis, MO). Sucrose was purchased from Pfanstiehl (Waukegan, IL). All the buffers and the mobile phase for SE-HPLC were prepared using deionized water from Water Pro PS Station (Labconco, Kansas City, MO) and were sterile filtered afterward using a 0.2 μm filter. CE-SDS gel separation buffer, SDS-MW sample buffer, 0.1N NaOH, 0.1N HCl, and MW size protein standards were purchased from Beckman Coulter. ß-mercapto-ethonal was purchased from Amresco (Solon, OH).

**Table 1:** Summary of Composition of the Individual and Mixture bnAb Formulations

bnAb	Protein Concentration (mg/mL)	ASu Formulation Buffer
3BNC117	104 ± 2	20 mM Acetate with 9% sucrose and 0.01% w/v polysorbate 80, pH 5.2
PGT121	121 ± 1	
3BNC117 + PGT121	117 ± 1	

The bnAb coformulation contained a 1:1 (w/v) mixture of each protein.

### Methods

#### PEG Precipitation Assay

The PEG precipitation assay was performed as previously described.^49^ Briefly, stock solutions of PEG-10,000 ranging from 0% to 40% (w/v) were prepared in 2 different buffers: (1) 20 mM acetate with 9% sucrose and 0.01%w/v polysorbate 80, pH 5.2 (ASu), and (2) 20 mM sodium phosphate, 150 mM sodium chloride pH 7.2 (PBS). Two hundred microliters of each PEG-10,000 solution was added to a 96-well polystyrene plate with filter at the bottom (Corning Life Sciences, Corning, NY) in triplicate. Individual or mixture bnAb solutions were diluted to 1 mg/mL, using either their respective formulation buffer or PBS, and then 50 μL of the diluted samples were added to the wells to achieve final protein concentrations of 0.2 mg/mL. The plates were incubated overnight at room temperature and then centrifuged at 3500 × *g* for 15 min. The filtrate was collected in a clear 96-well collection plate (Greiner Bio-One North America Inc., Monroe, NC). Two hundred microliters of the filtrate was transferred into a 96-well ultraviolet (UV) Star microplate (Grenier#655801) and the absorbance at 280 nm of each well was measured using SpectraMax M5 UV−visible plate reader (Molecular Devices LLC, Sunnyvale, CA). The concentration of each bnAb was calculated using their respective extinction coefficient (Table 1). The concentration of bnAb versus PEG-10,000 percentage was fit using a standard 4-parameter modified hill-slope sigmoidal curve equation (Eq. 1) using Python (x,y) version 2.7.6.0.

(1) $$y=b+\left(\frac{t-b}{1+e^{s\left(mid-x\right)}}\right)$$

Where, t = top plateau, b = bottom plateau, mid = x-axis midpoint, and s = slope. The %PEG_midpt_ values and apparent solubility value parameters were then calculated from the resulting curve fit as described in detail elsewhere.^49^

#### Differential Scanning Calorimetry

DSC thermograms for bnAbs were collected using an Auto-VP capillary differential scanning calorimeter (MicroCal/GE Health Sciences, Pittsburgh, PA). Thermograms were recorded from 10°C to 110°C at a scan rate of 1°C/min. The bnAb samples were prepared in 20 mM acetate with 9% sucrose and 0.01% w/v polysorbate 80, pH 5.2 at concentration of 0.1 mg/mL and the experiment was performed in triplicate. A buffer baseline was subtracted from each bnAb thermogram and the data were normalized using Microcal DSC software in Origin 7.0. The peaks were fitted using mathematical model fit in Origin 7.0 to calculate the values of thermal onset temperature (T_onset_) and thermal unfolding temperature (T_m_).

#### Real-Time and Accelerated Stability Study

For accelerated and real-time stability studies, 200 μL or 1 mL of each bnAb, either individually or in a 1:1 mixture, were dispensed into 1.0 mL or 2.0 mL (13 mm) glass vials and sealed with FluroTec-coated rubber stoppers and aluminum caps (West Pharmaceutical Services, Exton, PA). Vials containing protein or formulation buffer alone (in duplicate) were stored in the dark at up to 5 temperatures (−80°C, −20°C, 4°C, 25°C, 30°C, and 40°C) for up to 12 weeks. At each indicated time point, vials were visually inspected and then analyzed. For those vials containing a 1 mL sample volume, time points were analyzed by repeated sampling from a single vial.

### Neutralization Activity Assay

Neutralization titers of monoclonal Abs were determined using a luciferase-based assay in TZM.bl cells as previously described.^50^, ^51^ Briefly, mAb samples were tested using a primary concentration of 25 μg/mL with 5-fold serial dilutions against a panel of 10 HIV-1 Env pseudoviruses that were selected for being either 3BNC117 sensitive/PGT121 resistant or 3BNC117 resistant/PGT121 sensitive. This allowed for the measurement of neutralizing activity of single Abs in samples containing a mixture of both 3BNC117 and PGT121. Antibody titrations were incubated with HIV-1 Env pseudoviruses for 1 h at 37°C, and TZM.bl cells were then added in growth media containing DEAE-dextran at a final concentration of 11 μg/mL. Assay plates were incubated for 48 h at 37°C, 5% CO_2_, and luciferase reporter gene expression was measured using Bright-Glo luciferase reagent (Promega) and a Victor 3 luminometer (Perkin Elmer). Neutralization titers (50% and 80% inhibitory concentrations, IC50 and IC80, respectively) were calculated as the mAb concentration at which relative luciferase units (RLU) were reduced by 50% or 80% compared to RLU in virus control wells after subtraction of background RLU in cell control wells. All assays were performed in a laboratory meeting good current laboratory practice standards.

### Size Exclusion High-Performance Liquid Chromatography

Before SE-HPLC, bnAb samples were diluted 100-fold using their respective formulation buffers and then centrifuged at 14,000 × *g* for 5 min. Forty microliters of each sample was subjected to SE-HPLC using a Shimadzu HPLC system equipped with a photodiode array detector (Shimadzu, Columbia, MD) and a TSKgel G3000SWx1 stainless steel column and TSKgel SWxI guard column (Tosoh Biosciences, San Francisco, CA). The mobile phase consisted of 0.2 M sodium phosphate (pH 6.8) set at a flow rate of 0.7 mL/min. The column and autosampler were operated at 30°C and 4°C, respectively. Gel filtration standards (Biorad Laboratories, Hercules, CA) were used to ensure HPLC, column integrity, and separation efficiency. Protein elution was monitored using an absorbance of 214 and 280 nm, and protein peaks were quantified using the *LC Solution* software (Shimadzu Corporation) as described in detail elsewhere.^35^ In addition, the samples were subjected to SE-HPLC without the column attached to better determine if larger aggregates are present in the samples or if the sample binds to the column (i.e., sample recovery).

Monomer and dimer peak fractions were collected using a Dionex (Sunnyvale, CA) Ultimate 3000 system equipped with a Waters (Milford, MA) XBridge BEH 200A column and a mobile phase of 100 mM sodium phosphate, 250 mM sodium chloride, pH 6.8. Peak fractions were purified after SE-HPLC separation by collecting fractions shortly after traveling through the HPLC detector; the duration of fraction collection was determined manually by monitoring the real-time signal display using Chromeleon software (Thermo Scientific, Waltham, MA). Fractions were buffer exchanged into 20 mM sodium acetate pH 5.5 and concentrated using a Centricon spin concentrator with a 30 kDa molecular cutoff. To confirm purity of the collections, 5 μL was analyzed by SE-HPLC and overlayed with the starting material.

### CE Sodium Dodecyl Sulfate

Formulated monoclonal antibody solutions were diluted to 1 mg/mL with a mixture of 5.6% ß -ME in SDS-MW sample buffer. Samples were heated in Eppendorf tubes at 70°C for 10 min and then transferred to PCR vials. CE analysis was performed using a Beckman PA800 instrument equipped with a UV diode-array detector (Beckman Corporation, Brea, CA). A bare-fused capillary with a total length of 30.2 cm, effective length of 20.2 cm, and an inner diameter of 50 μm was used for separation. The running conditions are described in the Beckman Coulter manual for IgG Heterogeneity kit.

### Multiattribute Peptide Mapping Method

The workflow for the MAM involves characterizing a monoclonal antibody using MS2 to identify the modifications on the antibody using an MS2 capable MS (Thermo Scientific Q Exactive HF Biopharma). Next a Pinpoint workbook is created for each of the modification that will be monitored. Once the Pinpoint workbook is created, the MS data only need to be collected in MS1 mode or with an MS1 only MS. Initially, the PTMs on the 3BNC117 antibody and PGT121 were characterized using data acquired on a Thermo Scientific Q-Exactive HF Biopharma mass spectrometer. These data were searched with Biopharma Finder (Thermo Scientific).

#### Sample Preparation

The reduction, alkylation, and trypsin digestion of bnAb samples were performed as previously described in Rogers et al.^48^ and then analyzed as described below.

#### Reversed-Phase LC

A Thermo Vanquish Dual Column UPLC (Thermo Fischer Scientific, Waltham, MA) was used for these experiments. Mobile phase A contained 0.1% formic acid in water and mobile phase B contained 0.1% formic acid in 100% acetonitrile. The following LC conditions were used: flow rate 0.25 mL/min, column temperature 50°C during the separation, and the autosampler was kept at 6°C. For separation analysis, a nominal load of 2 μg of the digest, based on final sample concentration, was injected onto a Zorbax C18 300-SB, 300 A pore size, 1.8 mm particle, 2.1 mm × 150 mm column (Agilent). The gradient started at 1% B until 5 min, then increased gradually to 10% in 1 min. Next, the gradient was ramped up from 10% B to 35% B in 70 min. From 70 to 75 min, the %B was increased to 60%. At 75.1 min, the %B was reduced to 1% until minute 80. Total run time per sample was 80 min. The wash and re-equilibration pump was running in parallel to the gradient pump. The wash and re-equilibration pump employs 5 “saw tooth” ramps up to 90% B, holds at 90% B, and then ramps back down to 1% B.

#### Mass Spectrometry

The tryptic peptides were separated and monitored by RP-HPLC coupled to MS (Thermo Scientific Q Exactive HF BioPharma; Thermo Fisher Scientific, Waltham, MA). The MS capillary temperature was maintained at 250°C with an S-lens RF level at 50. The MS spectra collection was performed at 120,000 resolution in positive polarity mode with an AGC target of 3E6, maximum ion time of 200 ms, and a scan range of 300 to 1800 m/z between 5 to 70 min run time. Accurate mass measurement of the electrospray ionization was monitored using a lock mass of 391.2843 (diisooctyl phthalate). The MS2 spectra were acquired with the following conditions. The resolution was set at 15,000. One microscan was acquired with the AGC target set at 1e5. The maximum ion time was 250 ms. A top 5 method was used with the normalized collision energy set at 26. The charge exclusion was set to ignore unassigned charge ions and ions that had a charge greater than 8. The dynamic exclusion was set to 10 s.

#### Search Parameters

The raw files from the Q Exactive HF Biopharma mass spectrometer were searched using Biopharma Finder (Thermo Scientific). The MS2 spectra were searched against the antibody sequence and trypsin. The default search parameters were used except that the maximum peak width was increased to 3.2 min. A variable mass search was also performed from −58 to 162. Carboxymethylation was set as a static modification. CHO glycans, oxidation, deamidation, hydroxylation, glycation, H2O loss, and NH3 loss were set as variable modifications.

#### MAM Analysis of the Dimer and Monomer SEC Fractions

The enriched dimer and monomer fractions were prepared for MAM analysis as described above. The data were also acquired similarly. To determine if the dimer fraction was different from the monomer fraction, peptides from the variable region, from each molecule, were examined. The area for each peptide was compared to a constant domain peptide. Three variable domain peptides for 3BNC117 were compared to 5 constant domain peptides. Six variable domain peptides for PGT117 were compared to 5 constant domain peptides. Ratios were calculated for each fraction and then the average fold enrichment was calculated.

## Results

### Apparent Solubility and Conformational Stability of bnAbs

**Figure 1:**
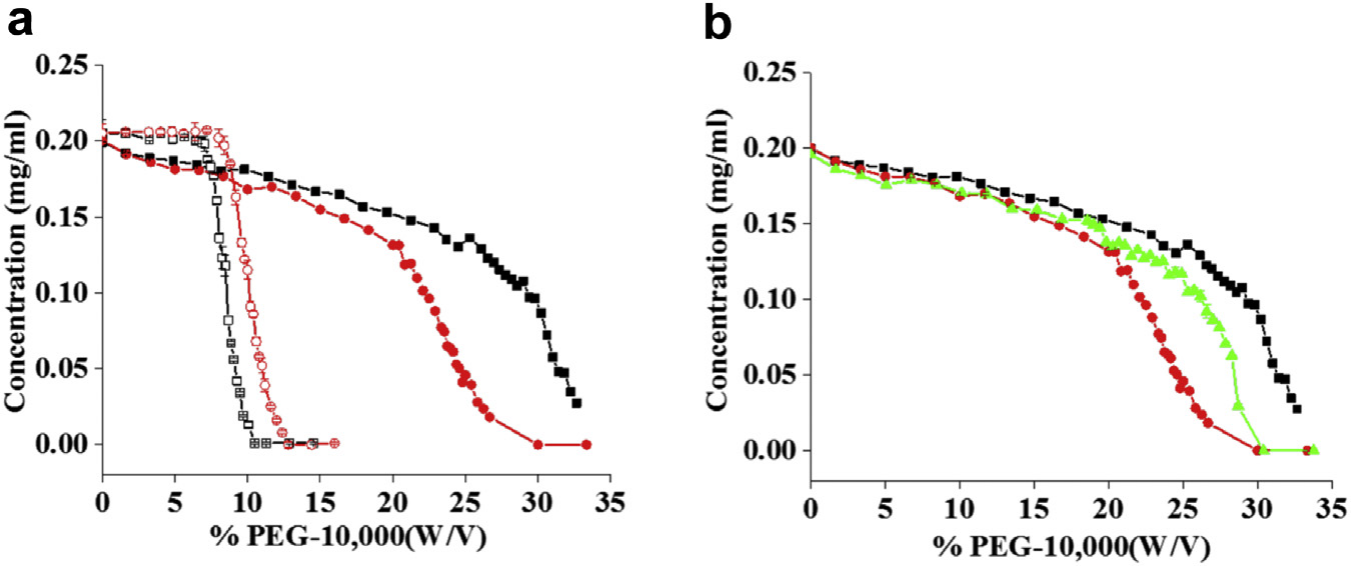
PEG precipitation of individual or 1:1 bnAb mixture formulated in PBS or ASu (see Methods for complete formulation compositions). PEG precipitation of (a) 3BNC117 and PGT121 in PBS and ASu formulation; (b) Individual or 1:1 mixture of 3BNC117 and PGT121 in ASu formulation. Error bars denote standard deviation from triplicate experiments. See Table 1 for composition of formulations. 3BNC117 at 100 mg/mL in ASu: solid black squares; 3BNC117 at 100 mg/mL in PBS: open black squares; PGT121 at 100 mg/mL in ASu: solid red circles; PGT121 at 100 mg/mL in PBS: open red circles; Coformulation of 3BNC117 and PGT121 each at 50 mg/mL in ASu: solid green triangles.

The relative apparent solubility profile of each bnAb sample was measured as a function of PEG concentration. For 3BNC117 and PGT121 in PBS buffer, a sigmoidal dose-response curve was observed for each bnAb (Fig. 1a). Using a 4-parameter modified hill-slope to fit both curves of 3BNC117 and PGT121 in PBS, % PEG midpoint values of 8.5% and 10.3% and relative apparent solubility values of approximately 33 and 55 mg/mL, respectively, were determined for the bnAbs 3BNC117 and PGT121 (Table 2). As also shown in Figure 1a, the %PEG versus protein concentration profiles of 3BNC117 in 2 different solutions (PBS buffer or ASu formulation) were distinct. A higher concentration of PEG was required to precipitate 3BNC117 in ASu compared to the bnAb in PBS, indicating a higher relative apparent solubility in the acetate-based formulations. Moreover, 3BNC117 in ASu did not completely precipitate out of solution at the highest PEG concentration tested (40% w/v). The PEG precipitation profile of 3BNC117 was also different between PBS buffer and the ASu formulation. The sigmoidal curve of the 3BNC117 bnAb as a function of PEG concentration was not observed when the protein was in ASu formulation. A gradual decrease in soluble protein was observed at lower PEG concentrations (0%-25%), which prevented the use of previously described models to fit the data. Therefore, the relative solubility profiles of 3BNC117 in PBS buffer versus ASu formulations were compared using the PEG concentration at which 50% of the protein had precipitated out of solution (PEG_midpt_). As shown in Table 2, the PEG_midpt_ value was higher for 3BNC117 in ASu (29% PEG) compared to the protein in PBS (8.5% PEG). The %PEG versus protein concentration profiles of PGT121 in both the PBS or ASu formulations showed overall similar trends to the observations of 3BNC117 with a few distinctions (Fig. 1a). The PGT171 displayed higher PEG midpoint and apparent solubility values in PBS buffer compared to 3BNC117 in PBS buffer but had lower PEG midpoint values, compared to 3BNC117, in the ASu formulation.

**Table 2:** Apparent Solubility and PEGmidpt Values of 2 Individual bnAb’s in Different Formulations, and the Mixture in One Formulation, as Measured by the PEG Precipitation Assay (*n* = 3)

bnAb	Formulation Buffer	%PEG_midpt_ (w/v)	Apparent Solubility (mg/mL)	
		Mean ± 1SD	Mean ± 1SD	*p* Value
3BNC117	PBS	8.5 ± 0.1	33 ± 3	<0.01^a^
	ASu	29.0 ± 0.0	N.D.	–
PGT121	PBS	10.3 ± 0.1	55 ± 6	–
	ASu	22.1 ± 0.0	N.D.	–
3BNC117 + PGT121	ASu	26.4 ± 0.0	N.D.	–

N.D., Could not be determined due to nonliner nature of experimental data (see text).

a Statistical significance compared to the PGT121 in PBS buffer with a *p* value <0.05, determined using student t-test.

Interestingly, the %PEG versus protein concentration profile of 2 bnAb’s in a 1:1 mixture in the ASu formulation was observed to be in between the values seen for the 2 individual proteins (Fig. 1b). Similarly, the corresponding PEG_midpt_ value of the mixture (26.4% PEG) was also in between the 2 individual bnAbs (22.1% and 29.0% PEG). Thus, the PEG precipitation assay distinguished between the 3 samples with the mixture behaving as a composite of the 2 individual components in the ASu formulation.

**Figure 2:**
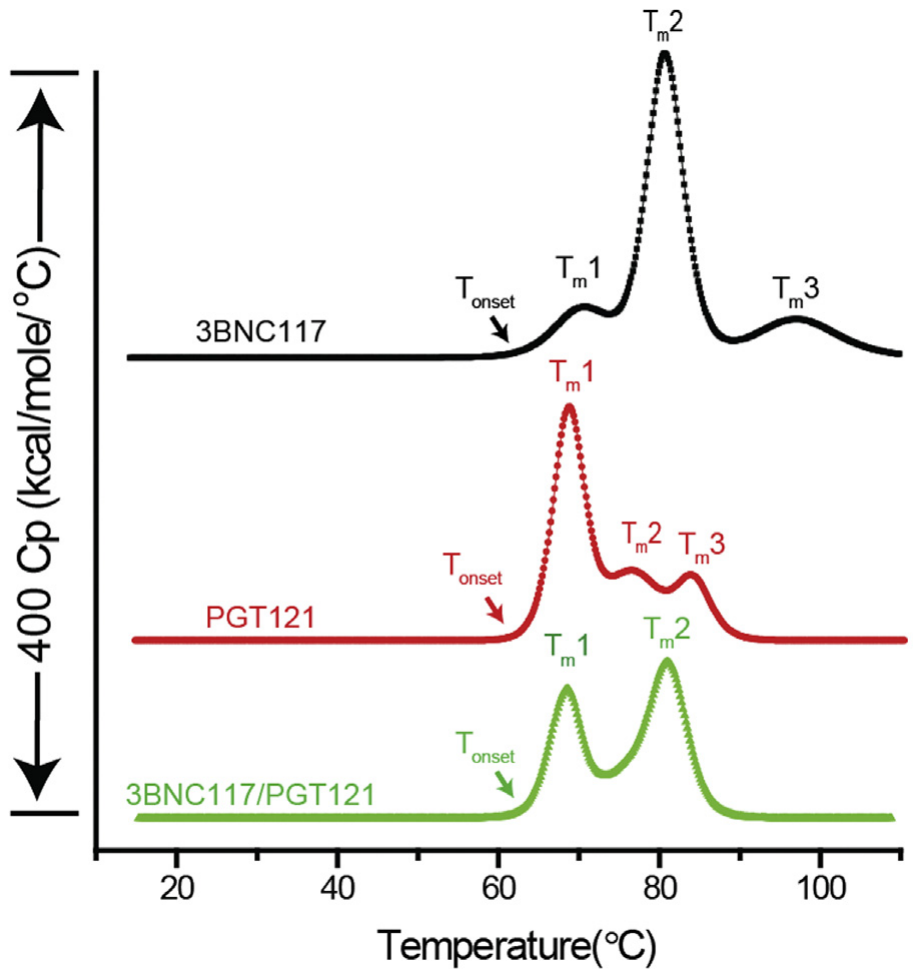
Representative DSC thermograms of individual or 1:1 bnAb mixture. See Table 1 for composition of formulations. See Table 3 for a summary of the thermal onset and thermal unfolding values calculated from the DSC thermograms. 3BNC117 at 100 mg/mL in ASu: solid black squares; PGT121 at 100 mg/mL in ASu: solid red circles; Coformulation of 3BNC117 and PGT121 each at 50 mg/mL in ASu: solid green triangles. DSC thermograms were offset for easier visualization.

DSC was used to examine the conformational stability of the 3 bnAb samples. Representative DSC thermograms comparing the individual bnAbs with the mixture are shown in Figure 2. Both bnAbs displayed at least 3 thermal unfolding events, with the major thermal melting transition temperatures (T_*m*_1, T_*m*_2, and T_*m*_3) having been shown previously to represent the unfolding of the C_H_2 domain, F_ab_, and C_H_3 domains, respectively, of IgG molecules.^52^, ^53^ Interestingly, a different and distinct profile of the thermograms was observed for each of the bnAb samples. First, the initial thermal transition, the onset unfolding temperature (T_onset_), was determined for each sample^54^, ^55^ as shown in Figure 2 and Table 3. The calculated T_onset_ value of the bnAb mixture was similar to PGT121 (∼61.3°C), which were both slightly lower than 3BNC117 (∼62.6°C). Second, we compared the 3 major thermal unfolding temperature values (T_*m*_1, T_*m*_2, and T_*m*_3) as shown in Figure 2 and Table 3. PGT121 showed a large initial unfolding transition (T_*m*_1) followed by 2 smaller transitions (T_*m*_2, T_*m*_3), whereas 3BNC117 displayed 1 small unfolding transition (T_*m*_1) followed by 1 large (T_*m*_2) and 1 small transition (T_*m*_3). The differences in the observed thermal profiles are likely attributed to structural differences between the 2 bnAbs. In the case of the bnAb mixture, 2 large and heterogeneous thermal transitions were observed, which appeared to overlap with the major transitions of the 2 individual bnAb thermograms (i.e., T_*m*_1 in PGT121-alone and T_*m*_2 in 3BNC117-alone). The calculated T_*m*_ values in the bnAb mixture, however, were somewhat different than 3BNC117- or PGT121-alone (Table 3), a difference possibly due to the contributions within the mixture of the minor transitions in the individual bnAbs. Furthermore, the 50% lower concentration of 3BNC117 in the bnAb mixture possibly resulted in the inability to quantify T_*m*_3 that was observed in 3BNC117-alone. Overall, since the T_onset_ and T_*m*_1 values reflect the initial structural alterations of the bnAb molecules versus temperature and are likely the most important in terms of assessing the stability of the proteins from a formulation point of view, 3BNC117 appeared slightly more conformational stable compared to PGT121 as a function of temperature, and mixing the 2 bnAbs together did not appear to notably effect the inherent stability (T_onset_ and T_*m*_1 values) of either protein.

**Table 3:** Conformational Stability of bnAbs Samples as Indicated by Thermal Onset Temperature Values (T_onset_) and Thermal Melting Temperature (T_m_1, T_m_2, and T_m_3) Values as Measured by DSC

bnAb	T_onset_ (°C)	T_m_1 (°C)	T_m_2 (°C)	T_m_3 (°C)
3BNC117	62.6 ± 0.4	70.6 ± 0.0	80.7 ± 0.0	97.1 ± 0.1
PGT121	61.3 ± 0.0	68.2 ± 0.0	76.2 ± 0.0	83.6 ± 0.0
3BNC117/PGT121	61.4 ± 0.3	68.6 ± 0.0	81.1 ± 0.0	–

See Table 1 for composition of each bnAb formulation.

See Figure 2 for representative thermograms for each bnAb formulation.

Results are presented as Avg ± SD.

All experiments were performed in triplicate.

### Real-Time and Accelerated Stability

The activity of the Abs was measured in the single and coformulated mixtures after 8 weeks of incubation. The data are presented in Table 4. To measure the functional neutralizing activity of each individual antibody in the mixture 2 virus panels were selected, 1 specific for the 3BNC117 bnAb and another representative panel specific for the PGT121 bnAb allowing us to interrogate the activity of each bnAb within the coformulated mixture without purifying the individual Abs. Values that are within 3-fold of the control are considered concordant by the assay validation criteria. A loss of activity would be an increase in the IC50 or IC80 greater than 3-fold of the control. The activity was corrected to account for the lower concentration of individual bnAbs in the mixture as compared to the singly formulated bnAbs. As evidenced by the data, both Abs and the mixtures show full activity after 8 weeks following storage for 8 weeks at 25°C and at 4°C.

**Table 4:** Neutralization Activity of 3BNC117, PGT121, and the 3BNC117/PGT121 Mixture to HIV Pseudovirus Panel Following 8 Weeks Incubation at Either 4°C or 25°C

3BNC117 Virus Sensitive Panel	Titer in TZM.bl Cells (μg/mL)									
	Q461.e2		C2101.c01		C4118.c09		R3265.c06		ZM249M.PL1	
	IC50	IC80	IC50	IC80	IC50	IC80	IC50	IC80	IC50	IC80
PGT121 4°C	>25	>25	>25	>25	>25	>25	>25	>25	>25	>25
3BNC117 4°C	0.051	0.167	0.020	0.106	0.036	0.155	0.079	0.692	0.028	0.129
PGT121/3BNC117 4°C	0.063	0.211	0.022	0.177	0.040	0.253	0.072	0.927	0.031	0.139
PGT121 25°C	>25	>25	>25	>25	>25	>25	>25	>25	>25	>25
3BNC117 25°C	0.039	0.136	0.015	0.084	0.032	0.136	0.117	0.921	0.031	0.134
PGT121/3BNC117 25°C	0.044	0.201	0.019	0.118	0.031	0.188	0.109	1.361	0.039	0.232
PGT121 Control	>25	>25	>25	>25	>25	>25	>25	>25	>25	>25
3BNC117 Control	0.057	0.196	0.028	0.115	0.052	0.215	0.204	1.513	0.055	0.243
PGT121 Virus Sensitive Panel	Titer in TZM.bl Cells (μg/mL)									
	1394C9G1 (Rev-)		ZM247v1 (Rev-)		Du422.1		377.v4.c9		Ce1172_H1	
	IC50	IC80	IC50	IC80	IC50	IC80	IC50	IC80	IC50	IC80
PGT121 4°C	0.759	7.011	0.011	0.049	0.013	0.053	0.322	1.099	0.005	0.020
3BNC117 4°C	>25	>25	>25	>25	>25	>25	>25	>25	>25	>25
PGT121/3BNC117 4°C	0.476	4.852	0.013	0.057	0.014	0.067	0.351	1.188	0.009	0.032
PGT121 25°C	0.721	6.606	0.019	0.063	0.011	0.055	0.283	0.991	0.005	0.017
3BNC117 25°C	>25	>25	>25	>25	>25	>25	>25	>25	>25	>25
PGT121/3BNC117 25°C	0.533	5.576	0.021	0.067	0.019	0.065	0.381	1.308	0.007	0.024
PGT121 Control	0.662	6.853	0.015	0.051	0.010	0.048	0.371	1.284	0.007	0.028
3BNC117 Control	>25	>25	>25	>25	>25	>25	>25	>25	>25	>25

Antibodies against a panel of viruses that included 5 3BNC117 sensitive/PGT121 resistant isolates and 5 3BNC117 resistant/PGT121 sensitive isolates (for specifically measuring 3BNC117 or PGT121, respectively).

All values are the average of duplicate measurements.

**Figure 3:**
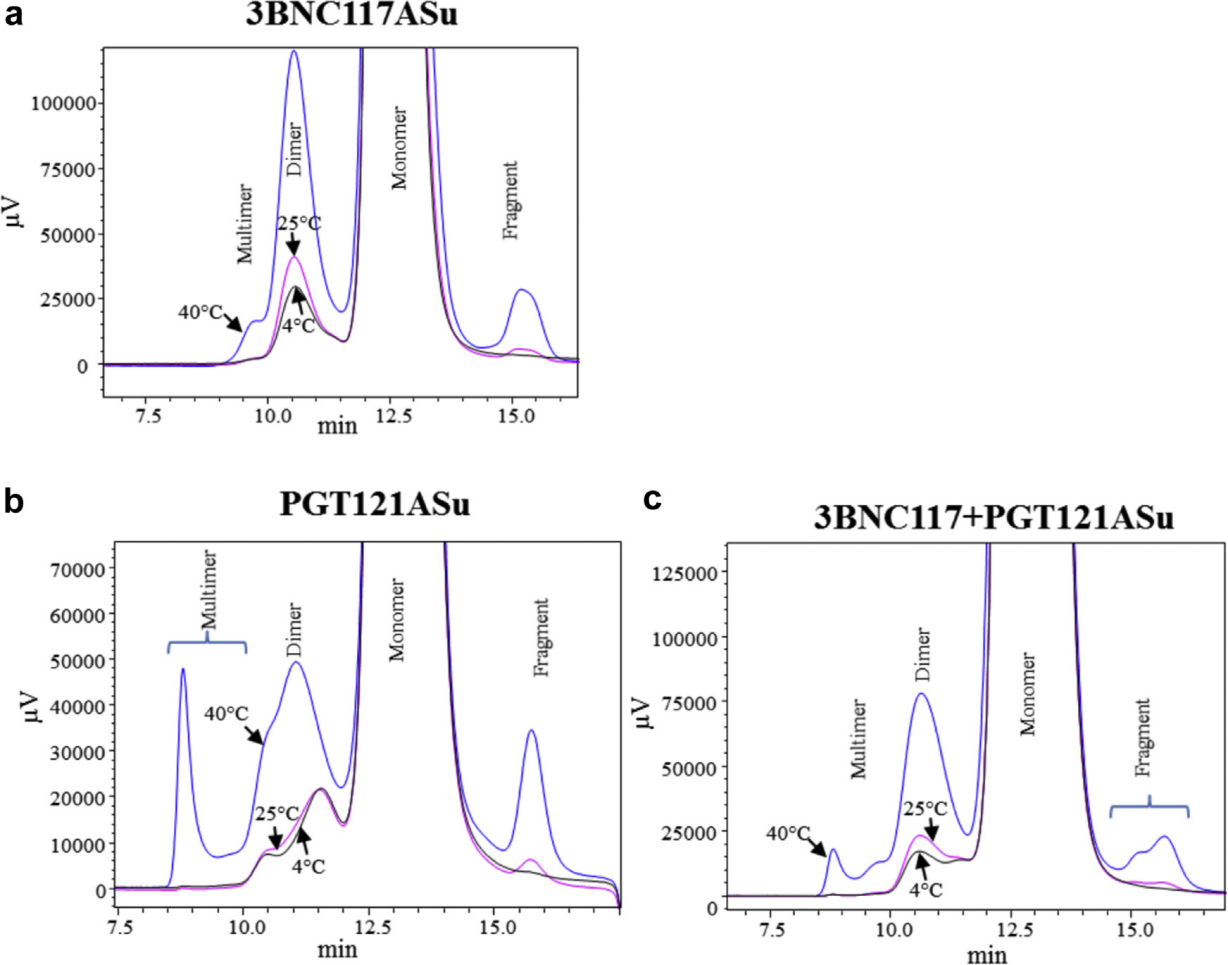
Representative SE-HPLC chromatograms of bnAbs monitored at 214 nm showing formation of aggregates and fragment species with respect to different temperatures (4°C–black; 25°C–pink; and 40°C–blue) at 12 weeks (a) 3BNC117ASu, (b) PGT121ASu, and (c) Mixture of 3BNC117 and PGT121 in ASu. See Table 1 for composition of formulations.

**Figure 4:**
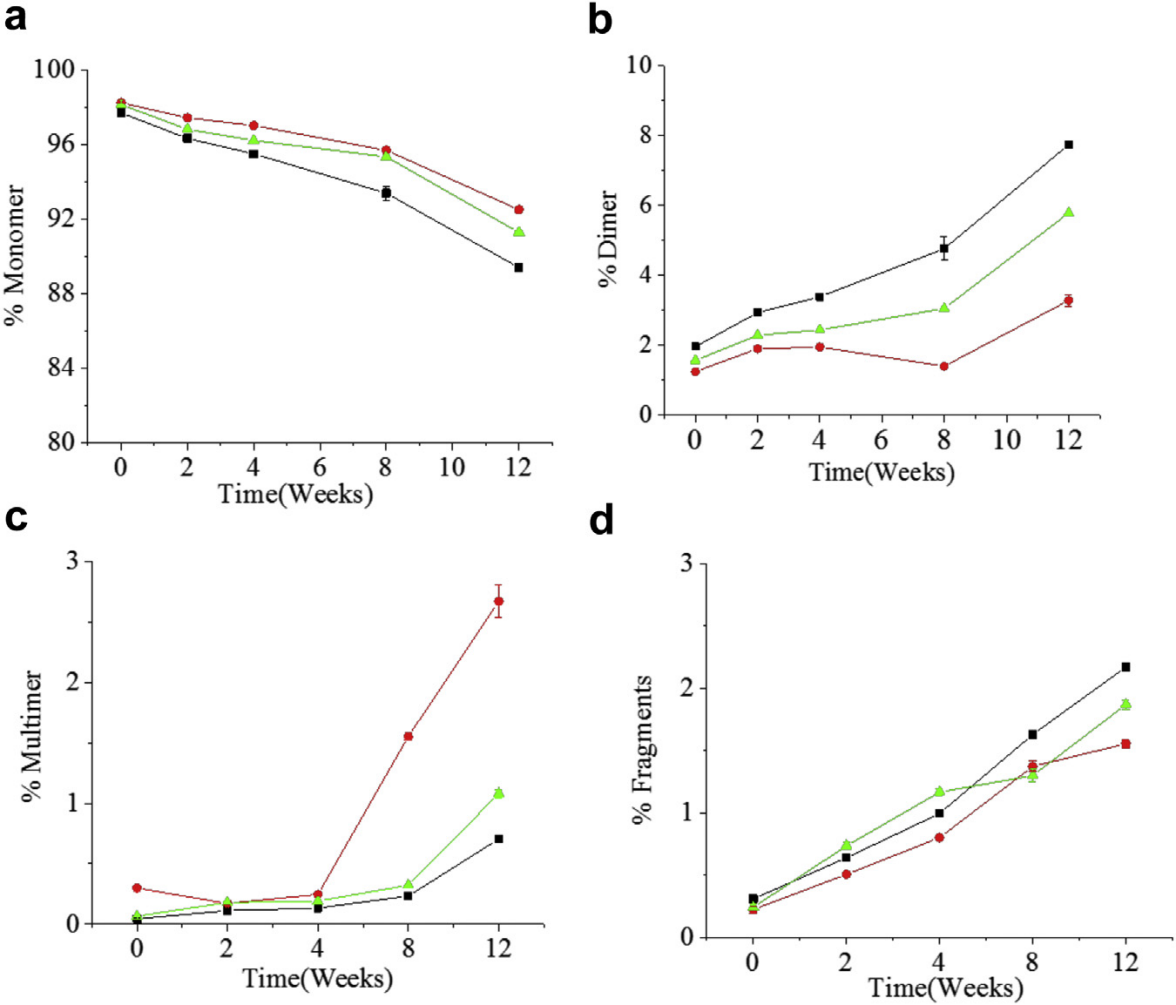
Percent amount of monomer, aggregates (dimers and multimers), and fragments as a function of time for bnAb formulations in ASu formulation following incubation at 40°C up to 12 weeks as measured by SE-HPLC. Data are plotted as mean ± SD with *n* = 4. See Table 1 for composition of formulations. 3BNC117 at 100 mg/mL in ASu: solid black squares; PGT121 at 100 mg/mL in ASu: solid red circles; Coformulation of 3BNC117 and PGT121 each at 50 mg/mL in ASu: solid green triangles. (a) Percent decrease in monomer vs. time, (b) percent dimer formation vs. time, (c) percent multimer formation vs. time, (d) percent fragmentation vs. time.

To better understand the aggregation propensity of the bnAbs during storage, individual bnAbs in ASu, and in a 1:1 mixture in ASu, were incubated at 5 temperatures (−80°C, −20°C, 4°C, 25°C, and 40°C) for up to 12 weeks. The oligomeric state of each sample was monitored using SE-HPLC and compared to a control (day 0). Representative chromatograms of 3BNC117 or PGT121 (individually and in a 1:1 mixture) after 12 weeks of incubation are shown in Figure 3. In addition to a monomer species, higher (e.g., dimers, multimers) and lower (e.g., fragments) molecular weight species were observed, which were more abundant at the higher incubation temperatures. The areas of each of these species observed in the SEC chromatograms were quantified and plotted relative to the Day 0 control. Each of the bnAb formulations contained primarily monomer with small levels of aggregates and fragments at time zero. The relative distribution of the individual species (monomer, dimer, etc.) did not change in the −80°C, −20°C, and 4°C samples over 12 weeks (data not shown). As expected, the loss of each bnAb monomer was accelerated at 40°C (Fig. 4a) compared to 25°C (Supplemental Fig. S1A), and a concurrent increase in the abundance of the higher and lower molecular weight species (Figs. 4b-4d and Supplemental Fig. S1B-S1D) was observed. After 12 weeks of incubation at 40°C, the relative monomer area decreased by ∼8% and ∼6% for 3BNC117 and PGT121 in ASu, respectively. Interestingly, although 3BNC117 primarily formed dimers during storage (∼6% increase compared to day 0 at 40°C), the PGT121 formed more complex multimer species (∼3% increase compared to day 0 at 40°C).

**Figure 5:**
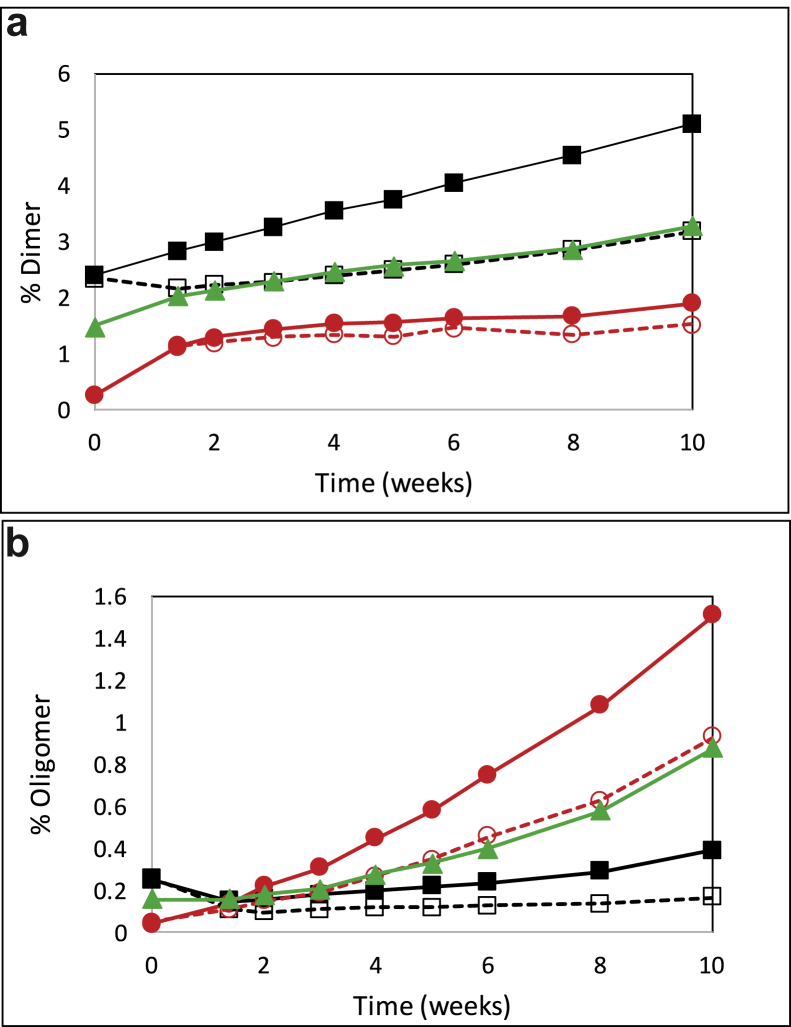
Dimer and oligomer (multimer) formation in individual bnAb formulations and in the coformulation following storage at 40°C. Formulations were as described in Table 1 and stored at 40°C for up to 12 weeks. SEC analysis was performed weekly. Dimer and oligomer were defined based on the elution time of the peaks compared to the main monomer peak. (a) Percent dimer versus time for all formulations; (b) % Oligomer versus time for all formulations. 3BNC117 at 100 mg/mL: solid black squares; 3BNC117 at 50 mg/mL: open black squares; PGT121 at 100 mg/mL: solid red circles; PGT121 at 50 mg/mL: open red circles; Coformulation of 3BNC117 and PGT121 each at 50 mg/mL: solid green triangles.

The relative monomer loss and the formation of multimers/dimers in the bnAb formulations alone and in mixtures were further investigated in an additional stability study in which each bnAb was formulated alone at 50 mg/mL and 100 mg/mL and together with both bnAbs coformulated at 50 mg/mL each in the mixture for a final total protein concentration of 100 mg/mL. Aliquots at each timepoint were removed from the same vial throughout the study to provide more precise quantitation of the aggregate species. As seen in Figure 5, the rates of dimer and multimer formation for the individually formulated bnAbs were severely reduced for both bnAbs when formulated at 50 mg/mL as compared to 100 mg/mL with 3BNC117 primarily forming dimer and PGT121 primarily forming oligomer at 40°C. In the mixture, the concentration of dimer at each timepoint was nearly identical to that of the individually formulated 3BNC117 at 50 mg/mL. Similarly, the concentration of oligomer at each timepoint matched that for the individually formulated PGT121 at 50 mg/mL suggesting that the aggregation of each bnAb in the mixture is independent of the other. At 30°C only dimer formation occurred with no discernable increase in oligomeric species for either antibody (data not shown).

**Figure 6:**
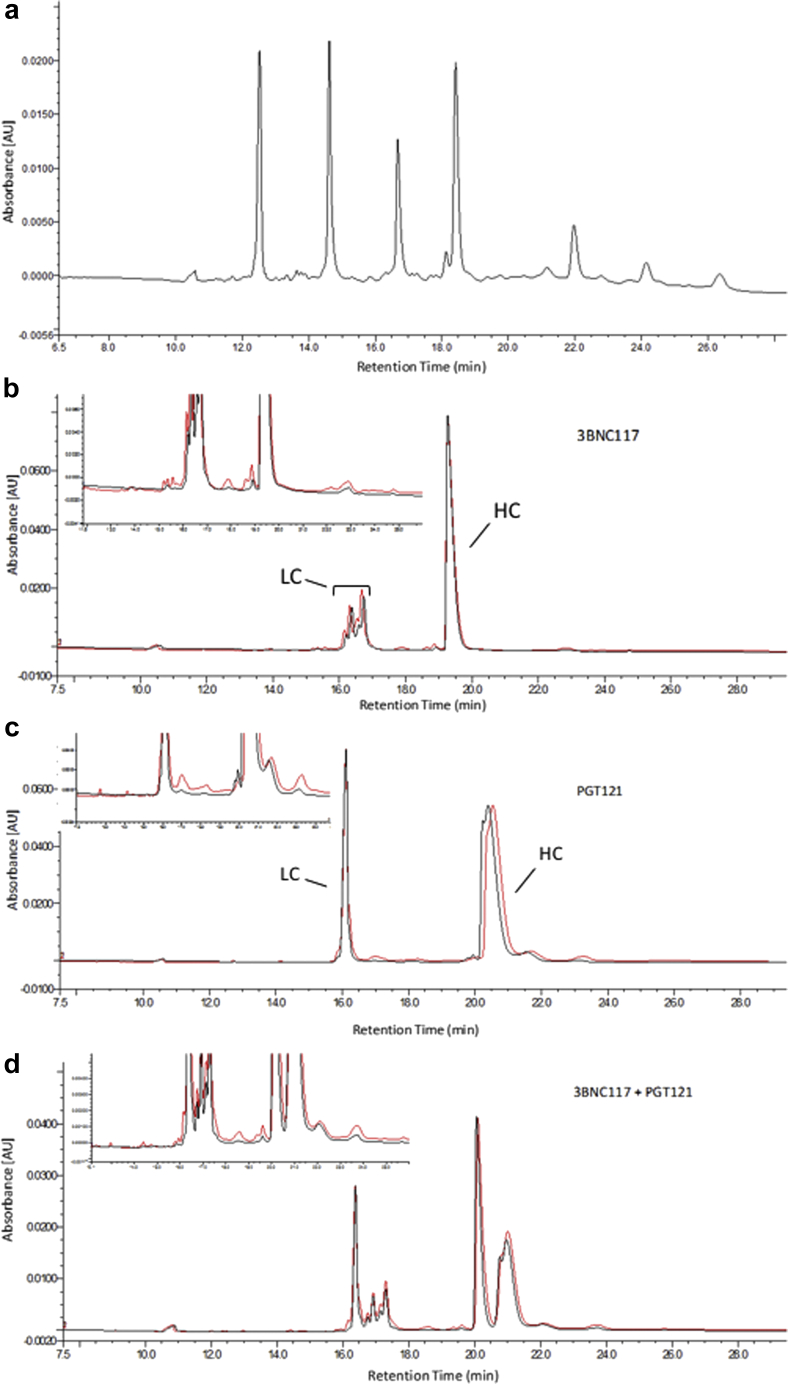
Quantification of clipped species in bnAb formulations in ASu from representative electropherograms as measured by reduced SDS CE. Formulations stored at 40°C for 12 weeks showed an increase in low molecular weight species migrating before the heavy chain and light chain peaks. Heterogeneity of the 3BNC117 light chain resulted in comigration of some degradation species with the main peaks in the coformulated samples. See Table 1 for composition of formulations. (a) Bio-Rad SDS-MW size standards: 10, 20, 35, 50, 100, 150, and 225 kDa, (b) 3BNC117, (c) PGT121, (d) 3BNC117 and PGT121 mixture. Black lines are for time 0 samples and red lines are after 12 weeks storage at 40°C.

**Figure 7:**
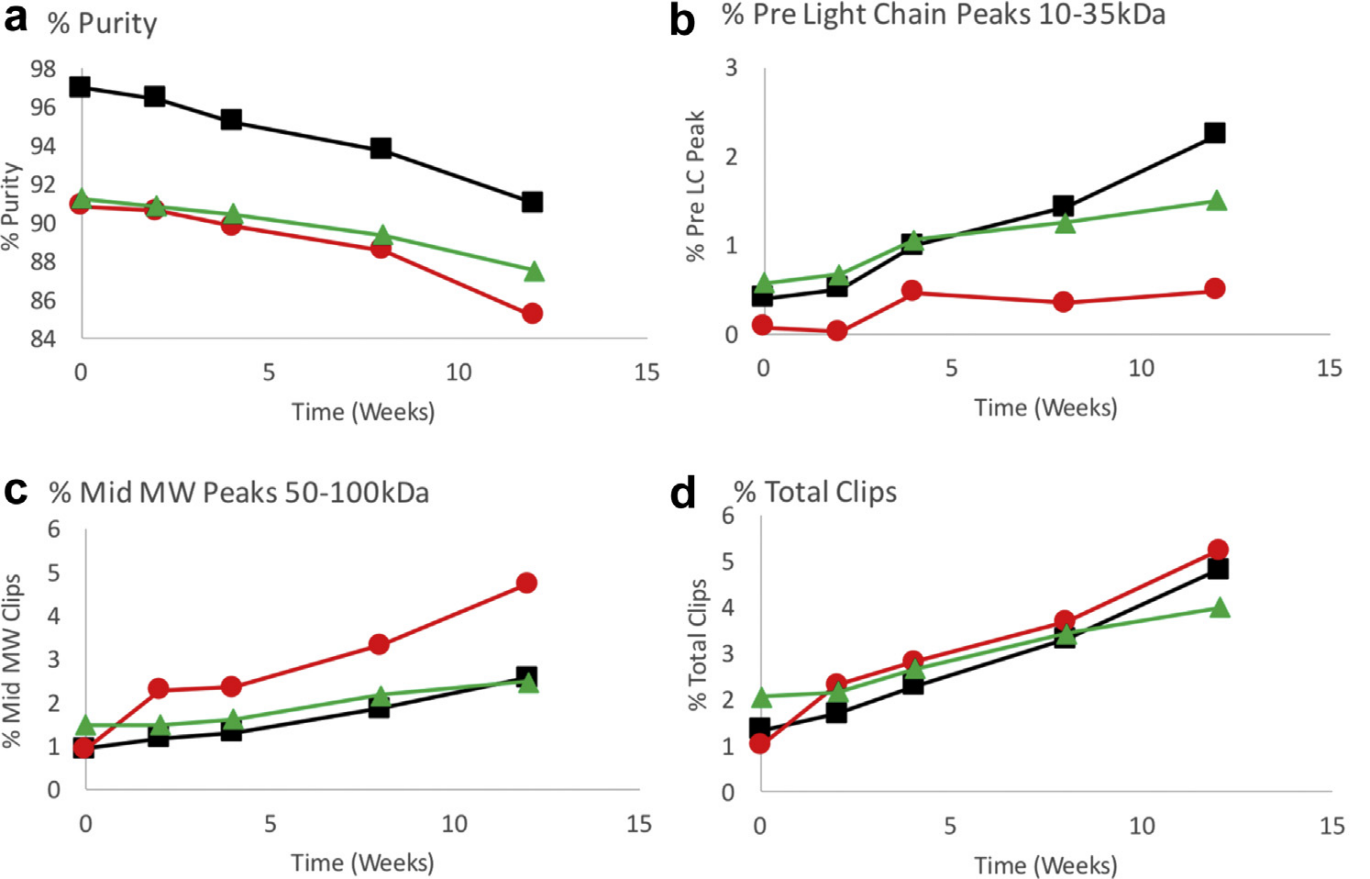
Quantification of clipped species formed over time in ASu formulations stored at 40°C as measured by reduced SDS CE. Low molecular weight species were grouped based on migration time relative to the heavy and light chain peaks, pre light chain species migrating before light chain, and mid molecular weight species migrating between the light and heavy chain peaks. Data plotted is based on a single analysis at each time point. 3BNC117 at 100 mg/mL: solid black squares; PGT121 at 100 mg/mL: solid red circles; Coformulation of 3BNC117 and PGT121 each at 50 mg/mL: solid green triangles. An error up to 0.7% was estimated using the standard error of the line regression analysis. See Table 1 for composition of formulations. (a) Percent purity vs. time, (b) percent pre light chains peaks, (c) percent mid molecular weight peaks, (d) percent total clips.

The amount of low molecular weight species was quantified for selected samples by monitoring the heavy chain (HC) and light chain (LC) peak purity by rCE-SDS. Representative electropherograms of 3BNC117, PGT121, and the 1:1 mixture are shown in Figure 6. Distinct differences are apparent for both the HC and LC profiles of the electropherograms between the singly formulated bnAbs. Heterogeneity of the 3BNC117 LC and the PGT121 HC resulted in multiple peaks in the time 0 control samples, which did not change over time. The heterogeneity is likely due to glycosylation, for example, 3BNC117 LC (Fig. 6a, 16-17 min) has a consensus glycosylation site at Asn KV:90 that is at least 90% occupied based on analysis by MAM (unpublished results). The PGT121 HC (Fig. 6b, 20-21 min) has 3 consensus glycosylation sequences, Asn HV:79, Asn HV:141, and Asn Fc-N:74. Based on MAM analysis, the Asn at HV:141 and Fc-N:74 are at least 99% occupied, whereas the site at Asn HV:79 is not occupied to any measurable degree. Clipped species of various molecular weights were observed at elevated temperature as compared with the time 0 control. Species were integrated in 3 groups (low, mid, and high MW) depending on the migration time relative to the LC and HC peaks. Low MW refers to species eluting earlier than the LC from 13-16 min, and mid MW refers to species eluting between the LC and HC at 17-19 min for 3BNC117 and 16.5-20 min between for PGT121, but not including the nonglycosylated species. The relative areas of each group were quantified and plotted as a function of incubation time in Figure 7and Supplemental Figure S2. Despite different purity levels at time 0, the rate of loss appears to be similar between the samples. Low MW clipped species form at a higher rate in the 3BNC117 sample compared with the PGT121 sample. The 1:1 mixture has a rate similar to the 3BNC117 sample. This trend was reversed in the mid MW clipped species where the PGT121 sample has a higher rate than the 3BNC117 and 1:1 mixture sample. However, clipped species observed in each of the single molecule formulations comigrate with the LC regions of the mixture. The low MW species between 15 and 16 min in the 3BNC117 sample comigrates with the LC of PGT121 in the 1:1 mixture sample. The mid MW species at 17 min in the PGT121 sample comigrates with the 3BNC117 LC in the mixture.

### MAM Data

**Figure 8:**
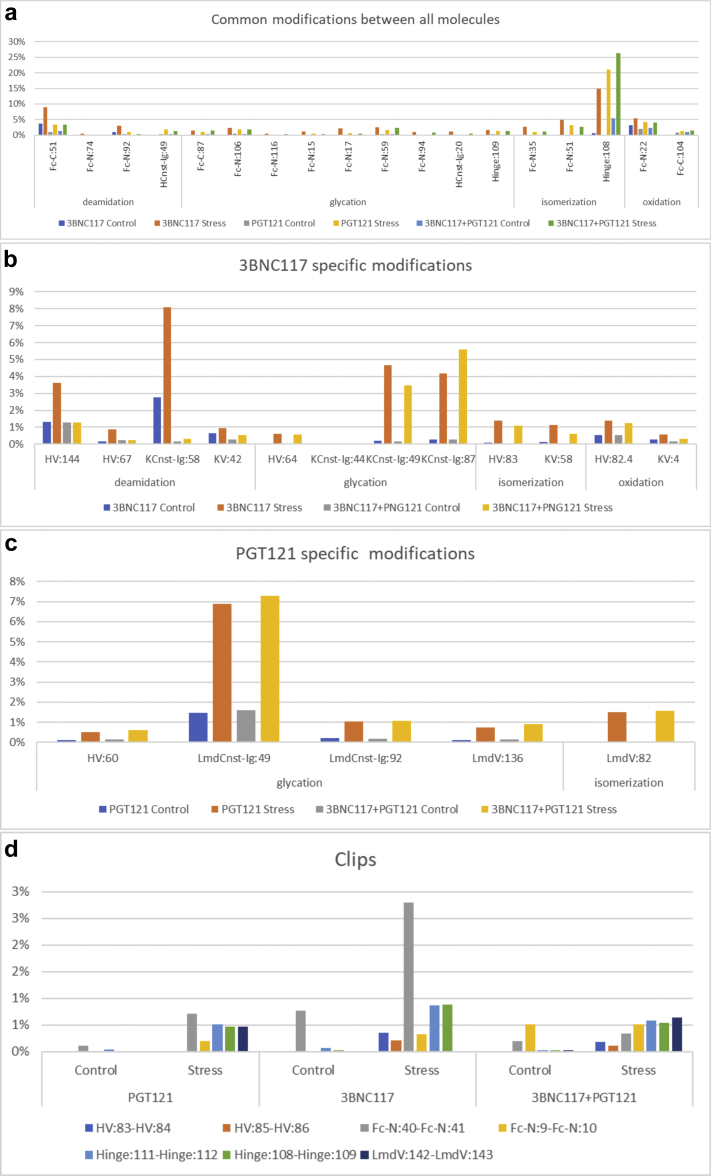
Comparison of relative quantification of posttranslational modifications and clips for 3BNC117, PGT121, and coformulated 3BNC117 + PGT121 at control conditions and stress conditions (12 wk at 40°C). (a) Comparison of deamidation, glycation, isomerization, and oxidation at common amino acid sites shared between each of the bnAb molecules/formulations. (b) Comparison of deamidation, glycation, isomerization, and oxidation at amino acid sites that are specific to 3BNC117. (c) Comparison of glycation and isomerization at amino acid sites that are specific to PGT121. (d) Comparison of clipping at common sites between all of the molecules (Fc-N:40-Fc-N:41, Fc-N:9-Fc-N:10, Hinge:111-Hinge:112, and Hinge:108-Hinge:109), sites specific to 3BNC117 (HV:83-HV:84 and HV:85-HV:86), and sites specific to PGT121 (LmdV:142-LmdV:143).

The primary sequence coverage as determined by the MAM mass spectrometry based analysis (see Methods) for the LC and HC of the single formulation of the 3BNC117 molecule were 96.1% and 96.7%, respectively. The primary sequence coverage for the LC and HC of the single formulation of the PGT121 molecule were 100% and 96.3%, respectively. The primary sequence coverage for the variable regions for the coformulated 3BNC117 and PGT121 was the same as the single formulations. The common PTMs, including deamidations, glycation, isomerization, and oxidation, observed between the single formulations and the coformulation are shown in Figure 8a. Low level deamidation, glycation, and oxidation occur within the common constant regions of the bnAb molecules. In addition, there are many aspartic acid residues that are common between 2 molecules, and 3 of the aspartic acid residues showed low levels of isomerization, whereas aspartic acid Hinge:108 is significantly isomerized in each formulation when the molecules are stressed. Figures 8b and 8c show specific modifications for 3BNC117 and PGT121, respectively. Glycation levels in both molecules increased on specific lysine residues when the molecules were incubated at 40°C. Low levels of aspartate isomerization and methionine oxidation were detected at specific sites on 3BNC117 and low levels of isomerization were detected at specific sites on PGT121. These modifications increased when the molecules were stressed. Deamidation of asparagine residues on 3BNC117 at sites HV:144, HV:67, KCnst-Ig:58, and KV:42 increased when the molecule was stressed. Interestingly, when 3BNC117 was coformulated with PGT121, the hot spots for Asn deamidation on 3BNC117 were less deamidated compared to the formulation of 3BNC117 alone. Finally, the detectable clips were quantified for the single formulations and the coformulation (Fig. 8d). All detectible clips increased when the molecules were stressed. The DP clip at FC-N:40-Fc-N-41 was the most abundant in each of the single formulations. When the molecules were coformulated, the clip at FC-N:40-Fc-N-41 was at a much lower level than was observed in the single formulations.

MAM analysis was also applied to characterize the dimer formed when each molecule was formulated separately or when the molecules were coformulated. Interestingly, when 3BNC117 was formulated alone, it formed more dimer when incubated at 40°C than PGT121 formulated alone and incubated at the same temperature (Fig. 5). The rate of dimer formation for 3BNC117 was 3.5X that of PGT121 based on the SEC analysis. MAM was applied to the dimer fractions and compared to the monomer fractions to determine if any modified forms of the molecules were enriched in the dimer fraction. No specific modifications were enriched in the dimer fraction of either 3BNC117 or PGT121 when formulated alone. When the coformulated sample was examined by MAM, a significant enrichment of 3BNC117-specific peptides was observed (Supplemental Fig. S3). Conversely, a decrease in the abundance of PGT121-specific peptides was observed in the dimer fraction. These data show that the dimer observed in the coformulated sample consists of approximately 3.5X more 3BNC117 than PGT121, a value similar to the increased rate of dimer formation for 3BNC117 as compared to PGT121.

## Discussion

Monoclonal Abs (mAbs) are widely used therapeutics for treatment of several human diseases including cancer and autoimmune diseases as well as for passive immunization. However, their effectiveness for the latter is limited against complex antigens/targets due to their mono-specificities for 1 antigen, which often develop drug resistance, for example, via viral mutations. As a result, approaches targeting multiple epitopes of the same antigen or different antigens are increasingly being developed to tackle drug resistance and to exhibit synergies in neutralizing and cell killing.^56^, ^57^ In addition, single shot injections of Abs (incorporating 2 or more mAbs in single injection) are advantageous, particularly in case where repeated injection of Abs are needed because they reduce the cost of administrative procedures and improve patient compliance by reducing the total number of injections.^31^ For example, pertuzumab and trastuzumab against HER2 (subdomain II and IV respectively) have shown complementarity in their actions in preclinical studies.^47^, ^58^ Several other codelivery approaches with monoclonal Abs have been explored in recent years.^59^ For instance, Symphogen A/S has developed Sym004, which is currently being evaluated in a clinical trial phase II as a mixture (1:1) of 2 chimeric IgG1 Abs (mAb992 and mAb1024 against nonoverlapping epitopes on EGFR to target metastatic colorectal cancer).^60^ In another study CL184 antibody cocktail, designed for postexposure prophylaxis against rabies, was found to be a more optimized product than the plasma-derived, polyclonal products obtained from rabies-vaccinated human donors or horses.^13^, ^61^, ^62^

Since 2010, several broadly neutralizing anti-HIV mAbs targeting different epitopes on the viral envelope glycoprotein (Env) have been isolated from HIV-positive patients^29^ and multiple clinical trials have now demonstrated the efficacy of the bnAbs in reducing viraema in patients.^18^, ^19^ More recently, studies in nonhuman primates have shown that combinations of these mAbs targeting different epitopes showed protection of animals from multiple SHIV challenges^63^ and supported the idea of using combinations of bnAbs as a prophylactic measure to significantly reduce the transmission of AIDS in vulnerable populations. For example, the Bill and Melinda Gates Foundation and its partners are exploring the use of this type of therapy for the Sub-Saharan Africa region. Doing so brings multiple challenges including cost, distribution, cold chains, and importantly, convenience for the patient. One way to increase convenience and help maintain adherence to the therapy by the patient is to combine the Abs in a single injection by manufacturing and providing the DP in a single vial as a coformulated product.

Coformulation of multiple proteins poses several formulation and analytical challenges. For example, the increased overall protein concentration, compared to an individual mAb, and differences in relative stability in a given formulation buffer, may lead to several pharmaceutical development challenges including enhanced attractive intermolecular protein–protein interactions, increased viscosity, and compromised structural integrity. These factors can increase the aggregation propensity of either/both molecules in a coformulation. Understanding aggregation propensity is a critical aspect during formulation development of biotherapeutics.^32^, ^64^ The long-term stability profile, including the aggregation propensity of the mAbs, are dependent on structural features of the individual mAb molecules. Hence, development of different analytical methods capable of assessing the stability of the mAb DPs after coformulation is a critical part of formulation development of comixtures. Assessing stability and aggregation propensity of individual mAb in a mixture poses several analytical challenges. Although numerous spectroscopy-based and separation-based analytical techniques are currently available for assessing protein aggregation,^65^ few reports are available on utility of these analytical techniques for studying the stability profile of complex antibody mixtures.^44^, ^45^

### Preformulation Characterization Studies With the Individual bnAbs and the Mixture

In this work, 2 broadly neutralizing Abs 3BNC117 and PGT121, targeting CD4bs and V3-glycan site, respectively, of the HIV gp120 viral protein, were coformulated. Following coformulation, the stability and degradation profiles of the individual Abs in the mixture were evaluated. As a first step, DSC and PEG precipitation assays were employed to evaluate the conformational stability and apparent solubility of individual bnAbs versus the bnAb mixture. Because solubility is a critical quality attribute in development of high concentration protein formulations, it is important to assess solubility in early stages of formulation design and development. The PEG precipitation-based methodology was used here for comparing relative solubility of monoclonal Abs alone and in mixtures. This assay allows for the rank ordering of Abs based on their relative solubility without requiring large quantities of protein.^49^ Interestingly, PEG_midpt_ value of the bnAbs mixture (26.4% PEG) was in between the 2 individual bnAbs (22.1% and 29.0% PEG), and thus the PEG precipitation assay was able to distinguish between the 3 samples with the mixture behaving as a composite of the 2 individual components. The lower solubility of the PGT121 may be due to an increase in exposed hydrophobic surfaces as compared to 3BNC117. For example, Standup Monolayer Affinity Chromatography differentiates molecules based on hydrophobicity^66^ showed a longer retention time for PGT121 as compared to 3BNC117 further supporting an increased exposure of hydrophobic surfaces for the antibody (data not shown).

Differential scanning calorimetry has been a widely utilized technique to characterize protein conformational stability during formulation development. It has also been used to analyze complex protein mixtures including human plasma and as a check of the physical stability of proteins in mixtures.^67^, ^68^ For example, in a study involving thermal denaturation of mixtures of α-lactalbumin (α-lac) and β-lactoglobulin (ß-lg), the mixture showed 2 thermal transitions at 37.5°C and 69.1°C (corresponding to the denaturation of apo-α-lac and ß-lg, respectively) with a shoulder at approximately 63°C (corresponding to the denaturation of holo- α -lac). Moreover, in a mixture, thermal transition temperature of apo-α -lac was increased and for ß -lg was decreased demonstrating that in a mixture thermal stability of α -lactalbumin increased while stability of ß -lactoglobulin decreased.^69^ In this study examining the DSC thermal profile of the bnAb mixture, 2 major transitions were observed, which were overlapped with major endothermic peak of individual bnAbs. In addition, the Tm values for each peak in the mixture were also correlated to Tm values of individual bnAbs. This result demonstrated that conformational stability of the bnAbs in the mixture is not significantly different from the individual bnAbs, and thus there are no notable destabilizing interactions between the 2 bnAbs when coformulated.

### Stability Studies With the Individual bnAbs and the Mixture to Monitor Aggregation Propensity

Aggregation is a major degradation pathway of mAbs, which is a concern during formulation development because aggregates can lower or increase potency as well as potentially trigger detrimental antidrug immunogenic responses on administration.^70^ Under stressed storage conditions, partial unfolding of antibody domains can occur, leading to self-association followed by nucleation and growth of aggregates.^71^ Both Fab and Fc structures within individual mAbs have aggregation-prone regions,^72^ and the overall rate of aggregation is also affected by solution conditions (e.g., pH, ionic strength, excipients) and the stress exposure conditions (e.g., temperature, agitation, freeze thaw).^73^, ^74^ Thus, a combination of external experimental conditions can affect aggregation propensities of Abs, as well as the intrinsic antibody properties (i.e., primary sequence and structural conformation).^71^ One of our primary interests in this study was to better understand the aggregation behavior of bnAbs when stored alone or in combination at elevated temperatures.

Size exclusion chromatography has been used extensively to monitor aggregation propensity and comparing stability of monoclonal Abs.^75^ Interestingly, a different aggregation profile was observed in the individual bnAbs. In addition, accelerated stability studies monitoring mAb dimer and multimer formation by SE-HPLC analysis showed that the 3BNC117 primarily formed dimer species, whereas PGT121 primarily formed multimer species at elevated temperatures (see Figure 4, Figure 5). Each of the species is in part due to nonreducible crosslinking as evidenced by the aggregation-related peaks in the reduced CE-SDS profiles. In the mixture, the rates of dimer and multimer formation were significantly lower than those expected for either protein at 100 mg/mL but were similar to the rates for the individual proteins at 50 mg/mL. This result suggests that the presence of each antibody does not influence the aggregation profile of the other antibody and that the proteins in the mixture aggregated as if they were formulated separately. In support of this possibility, the conformational and colloidal stability data from the DSC and PEG solubility studies as discussed above suggest the PGT121 is less soluble than 3BNC117. Isolation of the dimer species and interrogation by the MAM technique showed that the dimer was primarily 3BNC117 as would be expected if the proteins did not form coaggregates, albeit we cannot fully discard this hypothesis without additional experimentation. More work is underway to determine if the primary amino acid sequence or posttranslational modifications of 3BNC117 predisposes it to dimer formation and if the primary sequence of PGT121 predisposes it to multimerization.

The results of our studies are consistent with similar conclusions reached by Chen et al.^44^ for mixtures of multiple Abs stored under stress conditions. In their studies, 3 different Abs were mixed, heated to 70°C for 10 min, and the soluble fraction quantified by cation-exchange chromatography. Only one mAb precipitated while the other 2 remained soluble, and these results correlated to their melting temperature values. The authors concluded that “Our CEX52 results for the IgG1-A, IgG2-A, and IgG2-B mixture were surprisingly similar to the individual mAb data, suggesting little interference between the molecules.” Woodard et al.^45^ made similar observations in mixtures of up to 8 Abs.

**Figure 9:**
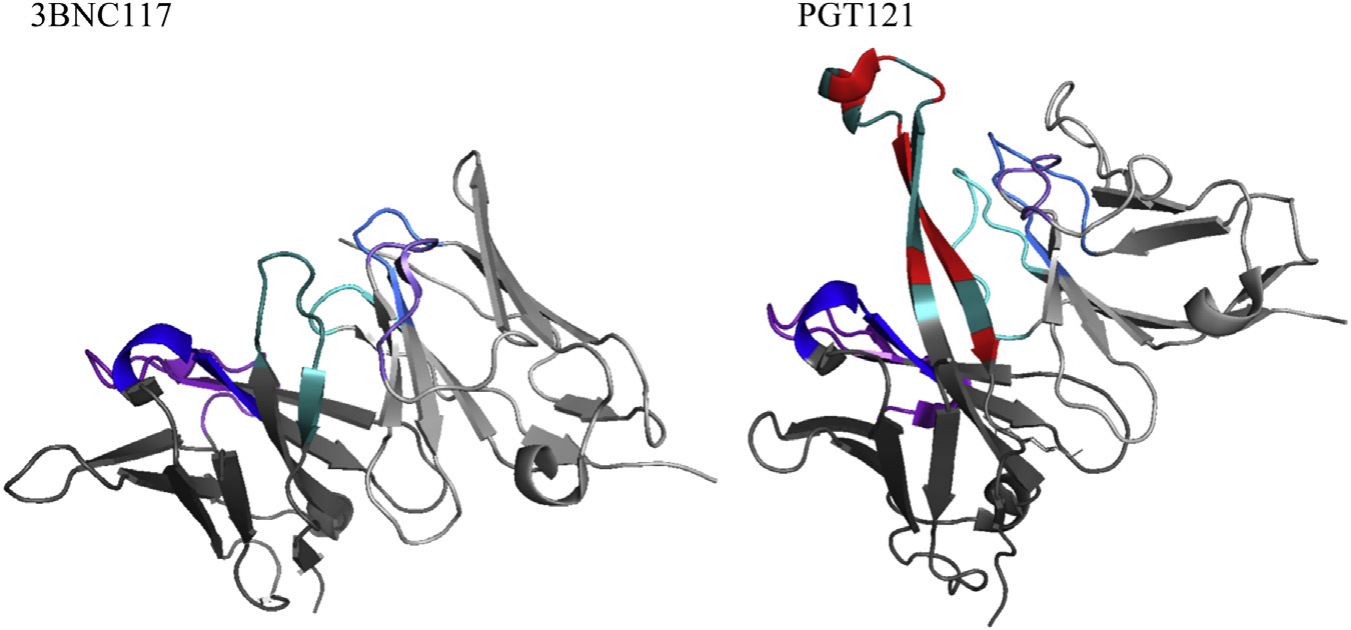
Structure models were generated from PDB files for 3BNC117^76^ (PDB ID: 4JPV) and PGT121^77^ (PDB ID: 4FQ1) using PyMol (The Pymol molecular graphics system, version 1.8 × Schrödinger, LLC) modeling software and show 3BNC117 and PGT121 Fv with highlighting of the CDR of the heavy and light chains. The heavy chain CDR3 is colored dark cyan. The hydrophobic residues of PGT121 HC_CDR3 are colored red. The rest of the coloring scheme is as follows: HC_CDR1 is blue, HC_CDR2 is dark orchid, HC_CDR3 is dark cyan, LC_CDR1 is dodger blue, LC_CDR2 is medium purple, and the LC_CDR3 is cyan. The sequence with annotations can be found in the supplementary material (Supplemental Figs. S4-S6).

Together, these data suggest that this may be a general phenomenon in mixtures of Abs and leading to the question of why the Abs do not form multimeric antibody complexes? For 3BNC117 and PGT121, the Fc regions are indistinguishable pointing toward the stability being driven by the Fab portions of the Abs. X-ray crystallographic structures clearly demonstrate differences between the Fv regions (Fig. 9) of the Abs such that structural interference may only allow association of like Abs and direct formation to either the dimer or multimer forms. PGT121 which formed primarily multimers, also had a much lower relative apparent solubility in PEG as compared to 3BNC117, suggesting the possible presence of an accessible hydrophobic patch on the surface that promotes protein–protein interactions. Comparison of PGT121 to 3BNC117 structures (Fig. 9) shows an extended CDR domain, HCDR3, on PGT121 forming a hydrophobic cluster with Phe at the tip and Trp and Tyr residues comprising the sides. The DSC data suggest a lower thermal stability for the PGT121 Fab. We hypothesize that at 40°C, the flexibility increases such that the hydrophobic residues lining the extended HCDR3 domain and possibly the LC/HC interface are more accessible and periodically reduces the steric hindrance of this region to the hydrophobic CDR spike allowing for interaction between neighboring proteins and forming a stable multimeric complex. Multimer formation by PGT121 at 30°C was not observed further suggesting that a thermal transition occurs between 30°C and 40°C for the protein leading to the increased flexibility. By this reasoning, 3BNC117 would not be expected to form multimers due to the increased enthalpic stability of its Fab region. If the lack of coaggregation holds for lower temperature storage over longer times, then this would also provide support to the hypothesis that antibody-antibody interactions have a degree of specificity, and that this specificity allows us to use data from singly formulated Abs to predict stability in coformulated solutions.

### Stability Studies With the Individual bnAbs and the Mixture to Monitor Chemical Stability

To further characterize the nature and levels of chemical modifications and degradation products, the MAM was employed. The MAM leverages high mass accuracy/high resolution MS data generated by Orbitrap technology and automated identification and relative quantification of posttranslational modifications with dedicated software.^48^ The bnAb samples were analyzed by MAM to quantify the formation of posttranslational modifications formed during storage at accelerated temperature. Chemical modifications such as isomerization, deamidation, oxidation, and glycation of proteins can play an important role in the protein aggregation pathway.^78^, ^79^ In this work, isomerization in the hinge region was the most abundant modification observed in each of the bnAb samples with the highest levels in the PGT121 samples. Aspartate residues in the hinge region are highly susceptible to isomerization under accelerated storage conditions, and it is favored when formulation conditions are mildly acidic.^80^ In contrast to PGT121, a relatively higher amount of oxidation and deamination was observed in 3BNC117. Interestingly, when 3BNC117 and PGT121 were coformulated, the amount of deamidation at HV:144 and KCnst-Ig:58, of 3BNC117, was significantly reduced. These data suggest that the asparagine residues at HV:144 and KCnst-Ig:58 are protected from deamidation in the coformulated samples. Each formulation was fractionated to enrich for the dimer/multimer and the monomer to better understand the possible role these PTMs play in aggregation. The PTM profiles for the single molecule formulations and the coformulation were similar in the dimer/multimer versus the monomer. Interestingly, as described above, the dimer/multimer fraction, in the coformulated sample, was enriched for 3BNC117-specific peptides. These data suggest that the dimer/multimer in coformulated sample is made up of mostly 3BNC117, and none of the PTMs monitored appear to be playing a role in the formation of the aggregates.

Another important aspect of protein stability is backbone hydrolysis, or clipping, which is typically quantified by SEC analysis under native-like conditions and by CE-SDS under reduced and denaturing conditions. Although SEC analysis shows an increase in clip species (see Fig. 4), this technique may underreport the total percent when only a single clip occurs, and the peptides are held in place by disulfide bonds. Reduced CE-SDS ameliorates this problem and allows for quantitation by UV detection. In the case of 3BNC117, the LC migrates anomalously due to glycosylation of Asn90, confounding the analysis when both PGT121 and 3BNC117 are coformulated. In that case, clips from the 3BNC117 LC can comigrate with the PGT121 LC. This potential problem can be circumvented by using the MAM to quantify the level of clipping in the sample. MAM analysis has the added benefit of defining the exact site(s) of hydrolysis for each of the Abs in the mixture. Although both molecules have similar sites of clipping, 3BNC117 shows increased susceptibility of the DP site between Fc:*N*-40 and Fc:*N*-41 when formulated individually but not in the coformulated mixture. The reason for this reduction is not clear and we are endeavoring to define the mechanism through which this may occur.

In addition, glycation was observed in the individual bnAb formulations as well as in the mixture when stored at higher temperature. Glycation is a condensation reaction between the aldehyde groups of reducing sugars and the primary or the secondary amines of proteins, in particular at lysine residues. In liquid formulations, sucrose is a commonly used protein stabilizer; however, it can hydrolyze into the reducing sugars glucose and fructose (especially at elevated temperatures and acidic solution pH), which can cause glycation in proteins. Glycation can affect protein structure, functionality, and therapeutic potential of proteins.^79^, ^81^ Therefore, characterization and quantification of glycation in protein-therapeutics is crucial. However, identification and characterization of glycation in therapeutic mAbs can be challenging due to multiple glycation prone sites. In individual bnAb as well as mixture samples, when stored at 40°C, glycation was observed in Fc region, hinge region, and LC constant region. More glycation sites of Lys specific in the Fc region were observed in 3BNC117 compared to PGT121. Low levels of glycation were observed in all of the samples stored at 4°C for the same period of time (data not shown). Glycation of Abs at higher temperatures has been reported in the previous studies when formulation buffer contained sucrose.^79^, ^81^ In a study by Gadgil et al., IgG formulations containing sucrose showed glycation in lysine residues when stored at 29°C and 37°C. Moreover, the rate of glycation increased incrementally with increasing temperature and time, and no glycation was observed in sample stored at 4°C for 18 months.^81^

## Conclusions

Although several recent studies demonstrated increasing needs for antibody combination formulations due to synergistic effects and improvements in efficacy, there is limited literature available on the pharmaceutical stability of individual Abs in coformulations. Such stability evaluation requires the use of stability-indicating analytical techniques able to differentiate individual Abs in a mixture. Our study provides insight into coformulation of 2 broadly neutralizing Abs and evaluated the physicochemical stability of coformulated Abs under both real time and stressed conditions. Many of the analytical methods employed in the study were able to monitor the structural integrity of both molecules within the mixture. The results demonstrated that in general, no physical interaction was observed between the 2 Abs in the mixture, but there were some notable effects on some of the Asn deamidation rates. Overall, the results presented herein highlight a case study of monitoring the physicochemical stability of monoclonal antibody mixtures and the power of combining multiple analytical techniques to cover key structural attributes of the molecules, which allows the screening of a codelivery system of Abs with desired stability characteristics. For example, the long term goal of providing stable, low cost liquid formulations containing a mixture of bnAbs for worldwide use will require ensuring stability during manufacturing, storage, transport, and administration, a similar stability challenge currently being addressed for vaccines distributed worldwide in the vaccine cold chain.^82^

## Supplementary Data

Supplemental Figure S1 Plots of changes in percent amount of monomer, aggregates and fragments as a function of time for bnAb formulations in ASu buffer following incubation at 25°C up to 12 weeks (a) % monomer (b) % dimer (c) % multimer (d) % fragments. The mean and standard deviation are based on 4 separate measurements. Data are plotted as mean± SD. See Table 1 for composition of the formulations. 3BNC117 at 100 mg/mL in ASu: solid black squares; PGT121 at 100 mg/mL in ASu: solid red circles; Co-formulation of 3BNC117 and PGT121 each at 50 mg/mL in ASu: solid green triangles.

Supplemental Figure S2 Quantification of clipped species formed over time in bnAb formulations stored at 25°C as measured by reduced SDS CE. Low molecular weight species were group based on migration time relative to the heavy and light chain peaks, pre light chain species migrating prior to light chain and mid molecular weight species migrating between the light and heavy chain peaks. Data plotted is based on a single analysis at each time point. 3BNC117 at 100 mg/mL: solid black squares; PGT121 at 100 mg/mL: solid red circles; Co-formulation of 3BNC117 and PGT121 each at 50 mg/mL: solid green triangles. An error up to 1% was estimated using the standard error of the line regression analysis. See Table 1 for composition of formulations.

Supplemental Figure S3 MAM analysis of the dimer fraction versus the monomer fraction in co-formulation. The variability for the mass spectrometry data are less than 10%. See Table 1 for composition of the formulations.

Supplemental Figure S4 3BNC117 kappa light chain sequence and numbering.

Supplemental Figure S5 PGT121 lambda light chain sequence and numbering.

Supplemental Figure S6 3BNC117 and PGT121 IgG1 heavy chain sequence and numbering.
