# Supplementary figures and images for: Coformulation of Broadly Neutralizing Antibodies 3BNC117 and PGT121: Analytical Challenges During Preformulation Characterization and Storage Stability Studies
Source: J Pharm Sci. 2018 Dec;107(12):3032–46. doi: 10.1016/j.xphs.2018.08.012 (PMC6269598; doi:10.1016/j.xphs.2018.08.012)

Supp Fig 1

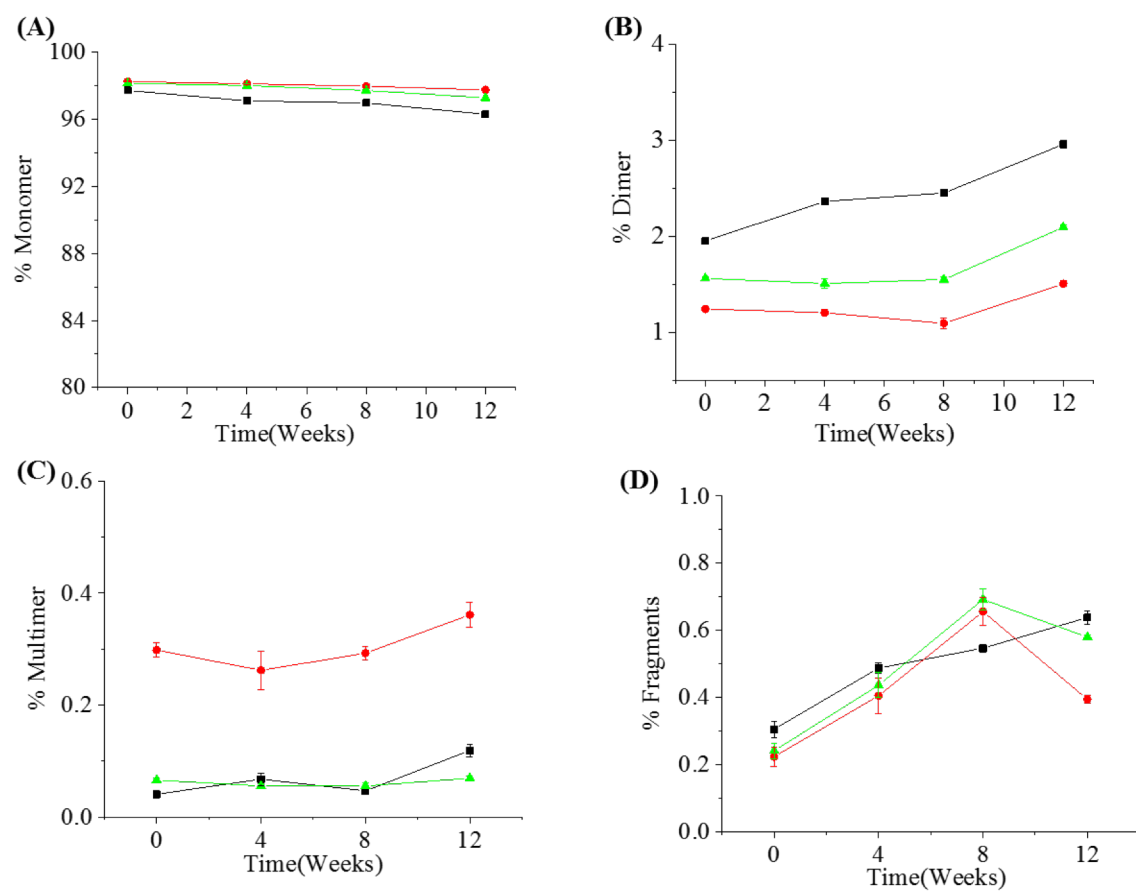

Supplement: Supplemental Figure S1 — Plots of changes in percent amount of monomer, aggregates and fragments as a function of time for bnAb formulations in ASu buffer following incubation at 25°C up to 12 weeks (a) % monomer (b) % dimer (c) % multimer (d) % fragments. The mean and standard deviation are based on 4 separate measurements. Data are plotted as mean± SD. See Table 1 for composition of the formulations. 3BNC117 at 100 mg/mL in ASu: solid black squares; PGT121 at 100 mg/mL in ASu: solid red circles; Co-formulation of 3BNC117 and PGT121 each at 50 mg/mL in ASu: solid green triangles. [file mmc1.pdf]

Supp Fig 2

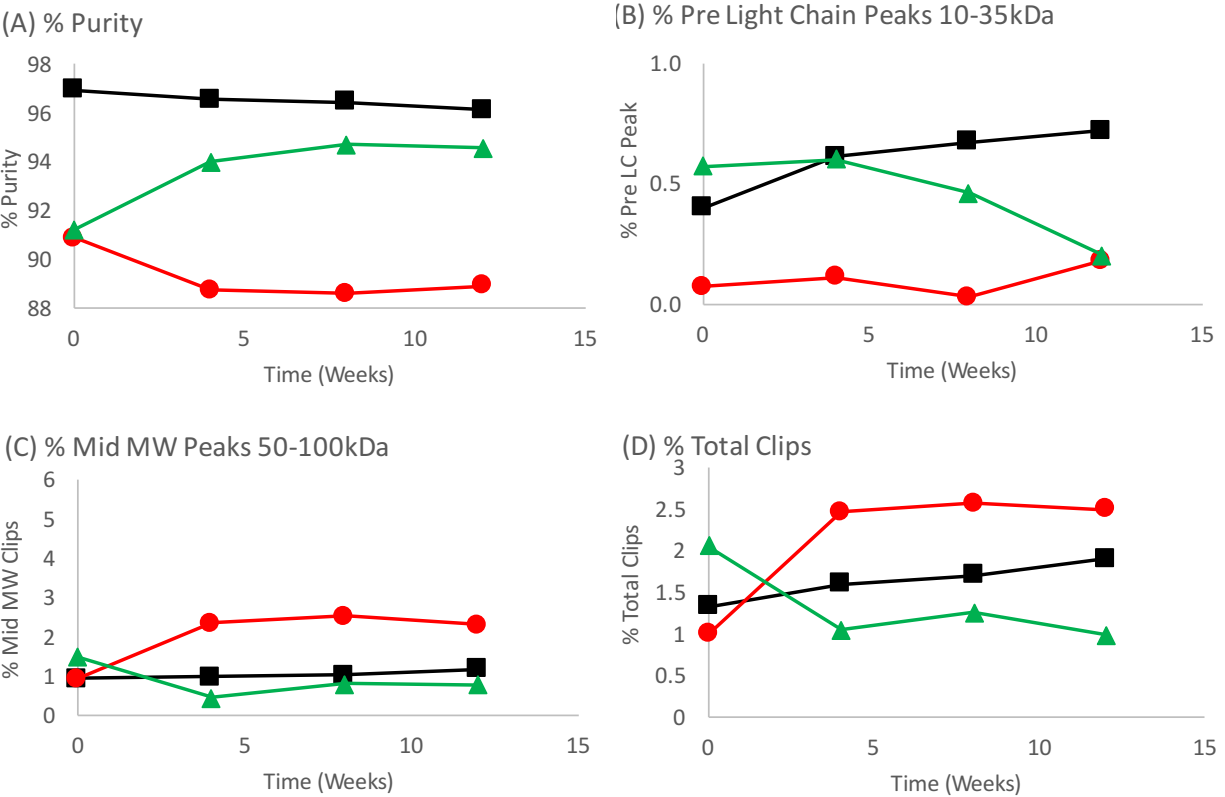

Supplement: Supplemental Figure S2 — Quantification of clipped species formed over time in bnAb formulations stored at 25°C as measured by reduced SDS CE. Low molecular weight species were group based on migration time relative to the heavy and light chain peaks, pre light chain species migrating prior to light chain and mid molecular weight species migrating between the light and heavy chain peaks. Data plotted is based on a single analysis at each time point. 3BNC117 at 100 mg/mL: solid black squares; PGT121 at 100 mg/mL: solid red circles; Co-formulation of 3BNC117 and PGT121 each at 50 mg/mL: solid green triangles. An error up to 1% was estimated using the standard error of the line regression analysis. See Table 1 for composition of formulations. [file mmc2.pdf]

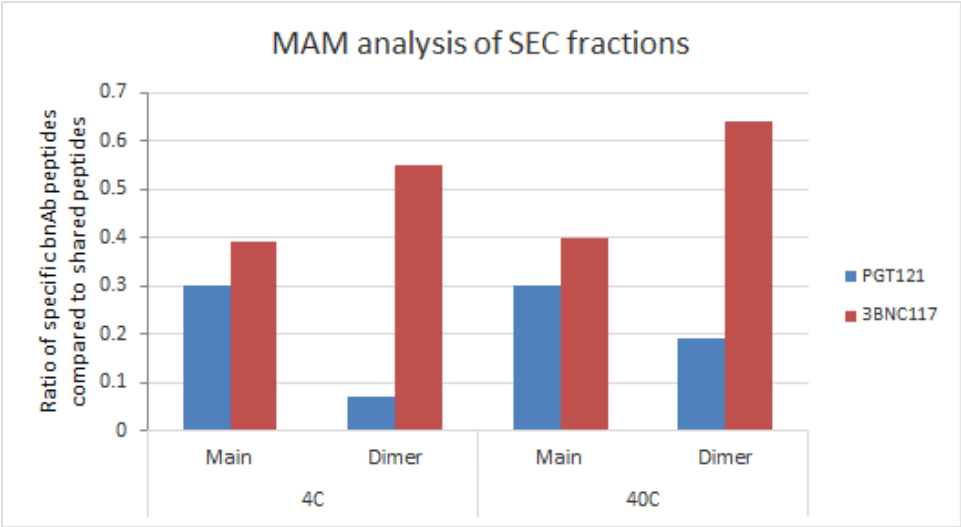

Supplement: Supplemental Figure S3 — MAM analysis of the dimer fraction versus the monomer fraction in co-formulation. The variability for the mass spectrometry data are less than 10%. See Table 1 for composition of the formulations. [file mmc3.pdf]
