# Supplementary material for: Coformulation of Broadly Neutralizing Antibodies 3BNC117 and PGT121: Analytical Challenges During Preformulation Characterization and Storage Stability Studies
Source: J Pharm Sci. 2018 Dec;107(12):3032–46. doi: 10.1016/j.xphs.2018.08.012 (PMC6269598; doi:10.1016/j.xphs.2018.08.012)
Supplement: Supplemental Figure S5 — PGT121 lambda light chain sequence and numbering. [file mmc5.pdf]

Supp Fig 5

ASN #	Ldr:-20	Ldr:-19	Ldr:-18	Ldr:-17	Ldr:-16	Ldr:-15	Ldr:-14	Ldr:-13	Ldr:-12	Ldr:-11	Ldr:-10	Ldr:-9	Ldr:-8	Ldr:-7	Ldr:-6	Ldr:-5	Ldr:-4	Ldr:-3	Ldr:-2	Ldr:-1	LmdV:1	LmdV:2	LmdV:3	LmdV:4	LmdV:5	LmdV:6	LmdV:7	LmdV:8	LmdV:9	LmdV:10	LmdV:11	LmdV:12	LmdV:13	LmdV:14	LmdV:15	LmdV:16	LmdV:17	LmdV:18	LmdV:19	LmdV:20	LmdV:21	LmdV:22	LmdV:23	LmdV:24	LmdV:25	LmdV:26	LmdV:27	LmdV:28	LmdV:29	LmdV:30	ASN #
	MiscLdr Sig																				LmdV FR1																							LmdV CDR1							
MatureLinear #																					0.1	0.2	0.3	0.4	0.5	0.6	0.7	0.8	1	2	3	4	5	6	7	8	9	10	11	12	13	14	15	16	17	18	18.1	18.2	18.3	19	MatureLinear #
PGT121_LC	M	Y	R	M	Q	L	L	S	C	I	A	L	S	L	A	L	V	T	N	S	-	-	-	-	-	-	-	-	S	D	I	S	V	A	P	G	E	T	A	R	I	S	C	G	E	K	-	-	-	S	PGT121_LC

ASN #	LmdV:31	LmdV:32	LmdV:33	LmdV:34	LmdV:35	LmdV:36	LmdV:37	LmdV:38	LmdV:39	LmdV:40	LmdV:41	LmdV:42	LmdV:43	LmdV:44	LmdV:45	LmdV:46	LmdV:47	LmdV:48	LmdV:49	LmdV:50	LmdV:51	LmdV:52	LmdV:53	LmdV:54	LmdV:55	LmdV:56	LmdV:57	LmdV:58	LmdV:59	LmdV:60	LmdV:61	LmdV:62	LmdV:63	LmdV:64	LmdV:65	LmdV:66	LmdV:67	LmdV:68	LmdV:69	LmdV:70	LmdV:71	LmdV:72	LmdV:73	LmdV:74	LmdV:75	LmdV:76	LmdV:77	LmdV:78	LmdV:79	LmdV:80	ASN #
	LmdV CDR1												LmdV FR2															LmdV CDR2															LmdV FR3								
MatureLinear #	20	21	22	22.1	22.2	22.3	22.4	22.5	23	24	25	26	27	28	29	30	31	32	33	34	35	36	37	38	39	40	41	42	42.1	42.2	42.3	42.4	42.5	42.6	42.7	42.8	43	44	45	46	47	48	49	50	51	52	53	54	55	56	MatureLinear #
PGT121_LC	L	G	S	-	-	-	-	-	R	A	V	Q	W	Y	Q	H	R	A	G	Q	A	P	S	L	I	I	Y	N	-	-	-	-	-	-	-	-	N	Q	D	R	P	S	G	I	P	E	R	F	S	G	PGT121_LC

ASN #	LmdV:80.1	LmdV:80.2	LmdV:80.3	LmdV:81	LmdV:82	LmdV:83	LmdV:84	LmdV:85	LmdV:86	LmdV:87	LmdV:88	LmdV:89	LmdV:90	LmdV:91	LmdV:92	LmdV:93	LmdV:94	LmdV:95	LmdV:96	LmdV:97	LmdV:98	LmdV:99	LmdV:100	LmdV:101	LmdV:102	LmdV:103	LmdV:104	LmdV:105	LmdV:106	LmdV:107	LmdV:108	LmdV:109	LmdV:110	LmdV:111	LmdV:112	LmdV:113	LmdV:114	LmdV:115	LmdV:116	LmdV:117	LmdV:118	LmdV:119	LmdV:120	LmdV:121	LmdV:122	LmdV:123	LmdV:124	LmdV:125	LmdV:126	LmdV:127	ASN #
	LmdV FR3																													LmdV CDR3																					
MatureLinear #	57	58	59	60	61	62	63	63.1	63.2	64	65	66	67	68	69	70	71	72	73	74	75	76	77	78	79	80	81	82	83	84	85	86	87	88	89	89.1	89.2	89.3	89.4	89.5	89.6	89.7	89.8	89.9	89.10	89.11	89.12	89.13	89.14	89.15	MatureLinear #
PGT121_LC	S	P	D	S	P	F	G	-	-	T	T	A	T	L	T	I	T	S	V	E	A	G	D	E	A	D	Y	Y	C	H	I	W	D	S	R	-	-	-	-	-	-	-	-	-	-	-	-	-	-	-	PGT121_LC

ASN #	LmdV:128	LmdV:129	LmdV:130	LmdV:131	LmdV:132	LmdV:133	LmdV:134	LmdV:135	LmdV:136	LmdV:137	LmdV:138	LmdV:139	LmdV:140	LmdV:141	LmdV:142	LmdV:143	LmdV:144	LmdV:145	LmdV:146	LmdV:147	LmdV:148	LmdV:149	LmdCnst-Ig:1	LmdCnst-Ig:2	LmdCnst-Ig:3	LmdCnst-Ig:4	LmdCnst-Ig:5	LmdCnst-Ig:6	LmdCnst-Ig:7	LmdCnst-Ig:8	LmdCnst-Ig:9	LmdCnst-Ig:10	LmdCnst-Ig:11	LmdCnst-Ig:12	LmdCnst-Ig:13	LmdCnst-Ig:14	LmdCnst-Ig:15	LmdCnst-Ig:16	LmdCnst-Ig:17	LmdCnst-Ig:18	LmdCnst-Ig:19	LmdCnst-Ig:20	LmdCnst-Ig:21	LmdCnst-Ig:22	LmdCnst-Ig:23	LmdCnst-Ig:24	LmdCnst-Ig:25	LmdCnst-Ig:26	LmdCnst-Ig:27	LmdCnst-Ig:28	ASN #
	LmdV CDR3											LmdV FR4											Lambda Constant Ig Domain																												
MatureLinear #	89.16	89.17	89.18	89.19	89.20	90	91	92	93	94	95	96	97	98	99	100	101	102	103	104	105	106	107	108	109	110	111	112	113	114	115	116	117	118	119	120	121	122	122.1	122.2	123	124	124.1	124.2	125	126	127	128	129	130	MatureLinear #
PGT121_LC	-	-	-	-	-	V	P	T	K	W	V	F	G	G	G	T	T	L	T	V	L	G	Q	P	K	A	A	P	S	V	T	L	F	P	P	S	S	E	-	-	E	L	-	-	Q	A	N	K	A	T	PGT121_LC

ASN #	LmdCnst-Ig:29	LmdCnst-Ig:30	LmdCnst-Ig:31	LmdCnst-Ig:32	LmdCnst-Ig:33	LmdCnst-Ig:34	LmdCnst-Ig:35	LmdCnst-Ig:36	LmdCnst-Ig:37	LmdCnst-Ig:38	LmdCnst-Ig:39	LmdCnst-Ig:40	LmdCnst-Ig:41	LmdCnst-Ig:42	LmdCnst-Ig:43	LmdCnst-Ig:44	LmdCnst-Ig:45	LmdCnst-Ig:46	LmdCnst-Ig:47	LmdCnst-Ig:48	LmdCnst-Ig:49	LmdCnst-Ig:50	LmdCnst-Ig:51	LmdCnst-Ig:52	LmdCnst-Ig:53	LmdCnst-Ig:54	LmdCnst-Ig:55	LmdCnst-Ig:56	LmdCnst-Ig:57	LmdCnst-Ig:58	LmdCnst-Ig:59	LmdCnst-Ig:60	LmdCnst-Ig:61	LmdCnst-Ig:62	LmdCnst-Ig:63	LmdCnst-Ig:64	LmdCnst-Ig:65	LmdCnst-Ig:66	LmdCnst-Ig:67	LmdCnst-Ig:68	LmdCnst-Ig:69	LmdCnst-Ig:70	LmdCnst-Ig:71	LmdCnst-Ig:72	LmdCnst-Ig:73	LmdCnst-Ig:74	LmdCnst-Ig:75	LmdCnst-Ig:76	LmdCnst-Ig:77	LmdCnst-Ig:78	ASN #
	Lambda Constant Ig Domain																																																		
MatureLinear #	131	132	133	134	135	136	137	138	139	140	140.1	140.2	141	142	143	144	145	146	147	147.1	148	149	150	151	152	153	154	155	156	157	158	159	160	161	162	163	164	165	166	166.1	166.2	166.3	166.4	166.5	167	168	169	170	171	172	MatureLinear #
PGT121_LC	L	V	C	L	I	S	D	F	Y	P	-	-	G	A	V	T	V	A	W	-	K	A	D	S	S	P	V	K	A	G	V	E	T	T	T	P	S	K	Q	-	-	-	-	-	S	N	N	K	Y	A	PGT121_LC

ASN #	LmdCnst-Ig:79	LmdCnst-Ig:80	LmdCnst-Ig:81	LmdCnst-Ig:82	LmdCnst-Ig:83	LmdCnst-Ig:84	LmdCnst-Ig:85	LmdCnst-Ig:86	LmdCnst-Ig:87	LmdCnst-Ig:88	LmdCnst-Ig:89	LmdCnst-Ig:90	LmdCnst-Ig:91	LmdCnst-Ig:92	LmdCnst-Ig:93	LmdCnst-Ig:94	LmdCnst-Ig:95	LmdCnst-Ig:96	LmdCnst-Ig:97	LmdCnst-Ig:98	LmdCnst-Ig:99	LmdCnst-Ig:100	LmdCnst-Ig:101	LmdCnst-Ig:102	LmdCnst-Ig:103	LmdCnst-Ig:104	LmdCnst-Ig:105	LmdCnst-Ig:106	LmdCnst-Ig:107	LmdCnst-Ig:108	LmdCnst-Ig:109	LmdCnst-Ig:110	LmdCnst-Ig:111	LmdCnst-Ig:112	LmdCnst-Ig:113	LmdCnst-Ig:114	LmdCnst-Ig:115	LmdCnst-Ig:116	LmdCnst-Ig:117	LmdCnst-Ig:118	LmdCnst-Ig:119	LmdCnst-Ig:120	LmdCnst-Ig:121	LmdCnst-Ig:122	LmdCnst-Ig:123	ASN #
	Lambda Constant Ig Domain																																													
MatureLinear #	173	174	175	176	177	178	179	180	181	182	183	184	184.1	185	186	187	188	189	189.1	189.2	190	191	192	193	194	195	196	197	198	199	200	200.1	200.2	201	202	203	204	205	205.1	206	207	208	209	210	211	MatureLinear #
PGT121_LC	A	S	S	Y	L	S	L	T	P	E	Q	W	-	K	S	H	R	S	-	-	Y	S	C	Q	V	T	H	E	G	S	T	-	-	V	E	K	T	V	-	A	P	T	E	C	S	PGT121_LC
