# Supplementary material for: Coformulation of Broadly Neutralizing Antibodies 3BNC117 and PGT121: Analytical Challenges During Preformulation Characterization and Storage Stability Studies
Source: J Pharm Sci. 2018 Dec;107(12):3032–46. doi: 10.1016/j.xphs.2018.08.012 (PMC6269598; doi:10.1016/j.xphs.2018.08.012)
Supplement: Supplemental Figure S6 — 3BNC117 and PGT121 IgG1 heavy chain sequence and numbering. [file mmc6.pdf]

Supp Fig 6

ASN #	Ldr:-20	Ldr:-19	Ldr:-18	Ldr:-17	Ldr:-16	Ldr:-15	Ldr:-14	Ldr:-13	Ldr:-12	Ldr:-11	Ldr:-10	Ldr:-9	Ldr:-8	Ldr:-7	Ldr:-6	Ldr:-5	Ldr:-4	Ldr:-3	Ldr:-2	Ldr:-1	HV:1	HV:2	HV:3	HV:4	HV:5	HV:6	HV:7	HV:8	HV:9	HV:10	HV:11	HV:12	HV:13	HV:14	HV:15	HV:16	HV:17	HV:18	HV:19	HV:20	HV:21	HV:22	HV:23	HV:24	HV:25	HV:26	HV:27	HV:28	HV:29	HV:30	ASN #
	MiscLdr Sig																				HV FR1																														
MatureLinear #																					1	2	3	4	5	6	7	7.1	8	9	10	11	12	13	14	15	16	17	18	19	20	21	22	23	24	25	26	26.1	27	28	MatureLinear #
3BNC117_HC	-	M	G	W	S	C	I	I	L	F	L	V	A	T	A	T	G	V	H	S	Q	V	Q	L	L	Q	S	-	G	A	A	V	T	K	P	G	A	S	V	R	V	S	C	E	A	S	G	-	Y	N	3BNC117_HC
MatureLinear #																					1	2	3	4	5	6	7	7.1	8	9	10	11	12	13	14	15	16	17	18	19	20	21	22	23	24	25	26	26.1	27	28	MatureLinear #
PGT121_HC	M	Y	R	M	Q	L	L	S	C	I	A	L	S	L	A	L	V	T	N	S	Q	M	Q	L	Q	E	S	-	G	P	G	L	V	K	P	S	E	T	L	S	L	T	C	S	V	S	G	-	A	S	PGT121_HC

ASN #	HV:31	HV:32	HV:33	HV:34	HV:35	HV:36	HV:37	HV:38	HV:39	HV:40	HV:41	HV:42	HV:43	HV:44	HV:45	HV:46	HV:47	HV:48	HV:49	HV:50	HV:51	HV:52	HV:53	HV:54	HV:55	HV:56	HV:57	HV:58	HV:59	HV:60	HV:61	HV:62	HV:63	HV:64	HV:65	HV:66	HV:67	HV:68	HV:69	HV:70	HV:71	HV:72	HV:73	HV:74	HV:75	HV:76	HV:77	HV:78	HV:79	HV:80	ASN #
		HV CDR1											HV FR2														HV CDR2																				HV FR3				
MatureLinear #	29	30	31	31.1	31.2	31.3	31.4	31.5	32	33	34	35	36	37	38	39	40	41	42	43	44	45	46	47	48	49	50	51	52	53	53.1	53.2	53.3	54	55	56	57	58	59	60	61	62	63	64	65	66	67	68	69	70	MatureLinear #
3BNC117_HC	I	R	D	-	-	-	-	-	Y	F	I	H	W	W	R	Q	A	P	G	Q	G	L	Q	W	V	G	W	I	N	P	-	-	-	K	T	G	Q	P	N	N	P	R	Q	F	Q	G	R	V	S	L	3BNC117_HC
MatureLinear #	29	30	31	31.1	31.2	31.3	31.4	31.5	32	33	34	35	36	37	38	39	40	41	42	43	44	45	46	47	48	49	50	51	52	53	53.1	53.2	53.3	53.4	54	55	56	57	58	59	60	61	62	63	64	65	66	67	68	69	MatureLinear #
PGT121_HC	I	S	D	-	-	-	-	-	S	Y	W	S	W	I	R	R	S	P	G	K	G	L	E	W	I	G	Y	V	H	K	-	-	-	-	S	G	D	T	N	Y	S	P	S	L	K	S	R	V	N	L	PGT121_HC

ASN #	HV:81	HV:82	HV:82.1	HV:82.2	HV:82.3	HV:82.4	HV:83	HV:84	HV:85	HV:86	HV:87	HV:88	HV:89	HV:90	HV:91	HV:92	HV:93	HV:94	HV:95	HV:96	HV:97	HV:98	HV:99	HV:100	HV:101	HV:102	HV:103	HV:104	HV:105	HV:106	HV:107	HV:108	HV:109	HV:110	HV:111	HV:112	HV:113	HV:114	HV:115	HV:116	HV:117	HV:118	HV:119	HV:120	HV:121	HV:122	HV:123	HV:124	HV:125	HV:126	ASN #
	HV FR3																																HV CDR3																		
MatureLinear #	71	72	73	74	75	76	77	78	79	80	81	82	83	84	85	86	87	88	89	90	91	92	93	94	95	96	97	98	99	100	101	102	103	104	105	106	106.1	106.2	106.3	106.4	106.5	106.6	106.7	106.8	106.9	106.10	106.11	106.12	106.13	106.14	MatureLinear #
3BNC117_HC	T	R	H	A	S	W	D	F	D	T	F	S	F	Y	M	D	L	K	A	L	R	S	D	D	T	A	V	Y	F	C	A	R	Q	R	S	D	-	-	-	-	-	-	-	-	-	-	-	-	-	-	3BNC117_HC
MatureLinear #	70	71	71.1	71.2	71.3	71.4	72	73	74	75	76	77	78	79	80	81	82	83	84	85	86	87	88	89	90	91	92	93	94	95	96	97	98	99	100	101	102	103	104	105	106	107	108	108.1	108.2	108.3	108.4	108.5	108.6	109	MatureLinear #
PGT121_HC	S	L	-	-	-	-	D	T	S	K	N	Q	V	S	L	S	L	V	A	A	T	A	A	D	S	G	K	Y	Y	C	A	R	T	L	H	G	R	R	I	Y	G	I	V	-	-	-	-	-	-	A	PGT121_HC

ASN #	HV:127	HV:128	HV:129	HV:130	HV:131	HV:132	HV:133	HV:134	HV:135	HV:136	HV:137	HV:138	HV:139	HV:140	HV:141	HV:142	HV:143	HV:144	HV:145	HV:146	HV:147	HV:148	HV:149	HCnst-Ig:1	HCnst-Ig:2	HCnst-Ig:3	HCnst-Ig:4	HCnst-Ig:5	HCnst-Ig:6	HCnst-Ig:7	HCnst-Ig:8	HCnst-Ig:9	HCnst-Ig:10	HCnst-Ig:11	HCnst-Ig:12	HCnst-Ig:13	HCnst-Ig:14	HCnst-Ig:15	HCnst-Ig:16	HCnst-Ig:17	HCnst-Ig:18	HCnst-Ig:19	HCnst-Ig:20	HCnst-Ig:21	HCnst-Ig:22	HCnst-Ig:23	HCnst-Ig:24	HCnst-Ig:25	HCnst-Ig:26	HCnst-Ig:27	ASN #
	HV CDR3												HV FR4											CH1																										
MatureLinear #	106.15	106.16	106.17	106.18	106.19	106.20	107	108	109	110	111	112	113	114	115	116	117	118	119	120	121	122	123	123.1	123.2	124	125	126	127	128	129	130	131	132	133	134	135	136	136.1	137	137.1	138	139	140	141	142	143	144	145	146	MatureLinear #
3BNC117_HC	-	-	-	-	-	-	Y	W	D	F	D	V	W	G	S	G	T	Q	V	T	V	S	S	-	-	A	S	T	K	G	P	S	V	F	P	L	A	P	-	S	-	S	K	S	T	S	G	G	T	A	3BNC117_HC
MatureLinear #	110	111	112	113	114	115	116	117	118	119	120	121	122	123	124	125	126	127	128	129	130	131	132	132.1	132.2	133	134	135	136	137	138	139	140	141	142	143	144	145	145.1	146	146.1	147	148	149	150	151	152	153	154	155	MatureLinear #
PGT121_HC	F	N	E	W	F	T	Y	F	Y	M	D	V	W	G	N	G	T	Q	V	T	V	S	S	-	-	A	S	T	K	G	P	S	V	F	P	L	A	P	-	S	-	S	K	S	T	S	G	G	T	A	PGT121_HC

ASN #	HCnst-Ig:28	HCnst-Ig:29	HCnst-Ig:30	HCnst-Ig:31	HCnst-Ig:32	HCnst-Ig:33	HCnst-Ig:34	HCnst-Ig:35	HCnst-Ig:36	HCnst-Ig:37	HCnst-Ig:38	HCnst-Ig:39	HCnst-Ig:40	HCnst-Ig:41	HCnst-Ig:42	HCnst-Ig:43	HCnst-Ig:44	HCnst-Ig:45	HCnst-Ig:46	HCnst-Ig:47	HCnst-Ig:48	HCnst-Ig:49	HCnst-Ig:50	HCnst-Ig:51	HCnst-Ig:52	HCnst-Ig:53	HCnst-Ig:54	HCnst-Ig:55	HCnst-Ig:56	HCnst-Ig:57	HCnst-Ig:58	HCnst-Ig:59	HCnst-Ig:60	HCnst-Ig:61	HCnst-Ig:62	HCnst-Ig:63	HCnst-Ig:64	HCnst-Ig:65	HCnst-Ig:66	HCnst-Ig:67	HCnst-Ig:68	HCnst-Ig:69	HCnst-Ig:70	HCnst-Ig:71	HCnst-Ig:72	HCnst-Ig:73	HCnst-Ig:74	HCnst-Ig:75	HCnst-Ig:76	HCnst-Ig:77	ASN #
	CH1																																																		
MatureLinear #	147	148	149	150	151	152	153	154	155	156	157	157.1	157.2	158	159	160	161	162	163	164	164.1	165	166	167	168	169	170	171	172	173	174	175	175.1	175.2	176	177	178	179	180	181	181.1	181.2	181.3	181.4	181.5	182	183	184	185	186	MatureLinear #
3BNC117_HC	A	L	G	C	L	V	K	D	Y	F	P	-	-	E	P	V	T	V	S	W	-	N	S	G	A	L	T	S	G	V	H	T	-	-	F	P	A	V	L	Q	-	-	-	-	-	S	S	G	L	Y	3BNC117_HC
MatureLinear #	156	157	158	159	160	161	162	163	164	165	166	166.1	166.2	167	168	169	170	171	172	173	173.1	174	175	176	177	178	179	180	181	182	183	184	184.1	184.2	185	186	187	188	189	190	190.1	190.2	190.3	190.4	190.5	191	192	193	194	195	MatureLinear #
PGT121_HC	A	L	G	C	L	V	K	D	Y	F	P	-	-	E	P	V	T	V	S	W	-	N	S	G	A	L	T	S	G	V	H	T	-	-	F	P	A	V	L	Q	-	-	-	-	-	S	S	G	L	Y	PGT121_HC

ASN #	HCnst-Ig:78	HCnst-Ig:79	HCnst-Ig:80	HCnst-Ig:81	HCnst-Ig:82	HCnst-Ig:83	HCnst-Ig:84	HCnst-Ig:85	HCnst-Ig:86	HCnst-Ig:87	HCnst-Ig:88	HCnst-Ig:89	HCnst-Ig:90	HCnst-Ig:91	HCnst-Ig:92	HCnst-Ig:93	HCnst-Ig:94	HCnst-Ig:95	HCnst-Ig:96	HCnst-Ig:97	HCnst-Ig:98	HCnst-Ig:99	HCnst-Ig:100	HCnst-Ig:101	HCnst-Ig:102	HCnst-Ig:103	HCnst-Ig:104	HCnst-Ig:105	HCnst-Ig:106	HCnst-Ig:107	HCnst-Ig:108	HCnst-Ig:109	HCnst-Ig:110	HCnst-Ig:111	HCnst-Ig:112	HCnst-Ig:113	HCnst-Ig:114	HCnst-Ig:115	HCnst-Ig:116	HCnst-Ig:117	HCnst-Ig:118	HCnst-Ig:119	HCnst-Ig:120	HCnst-Ig:121	HCnst-Ig:122	HCnst-Ig:123	Hinge:1	Hinge:2	Hinge:3	Hinge:4	ASN #
	CH1																																														Hinge				
MatureLinear #	187	188	189	190	191	192	193	194	195	196	197	198	199	199.1	200	201	202	203	203.1	203.2	203.3	204	205	206	207	208	209	210	211	212	213	214	214.1	214.2	215	216	217	218	219	219.1	220	221	221.1	221.2	221.3	221.4	221.5	221.6	221.7	221.8	MatureLinear #
3BNC117_HC	S	L	S	S	V	V	T	V	P	S	S	S	L	-	G	T	Q	T	-	-	-	Y	I	C	N	V	N	H	K	P	S	N	-	-	T	K	V	D	K	-	K	V	-	-	-	-	-	-	-	-	3BNC117_HC
MatureLinear #	196	197	198	199	200	201	202	203	204	205	206	207	208	208.1	209	210	211	212	212.1	212.2	212.3	213	214	215	216	217	218	219	220	221	222	223	223.1	223.2	224	225	226	227	228	228.1	229	230	230.1	230.2	230.3	230.4	230.5	230.6	230.7	230.8	MatureLinear #
PGT121_HC	S	L	S	S	V	V	T	V	P	S	S	S	L	-	G	T	Q	T	-	-	-	Y	I	C	N	V	N	H	K	P	S	N	-	-	T	K	V	D	K	-	K	V	-	-	-	-	-	-	-	-	PGT121_HC

ASN #	Hinge:5	Hinge:6	Hinge:7	Hinge:8	Hinge:9	Hinge:10	Hinge:11	Hinge:12	Hinge:13	Hinge:14	Hinge:15	Hinge:16	Hinge:17	Hinge:18	Hinge:19	Hinge:20	Hinge:21	Hinge:22	Hinge:23	Hinge:24	Hinge:25	Hinge:26	Hinge:27	Hinge:28	Hinge:29	Hinge:30	Hinge:31	Hinge:32	Hinge:33	Hinge:34	Hinge:35	Hinge:36	Hinge:37	Hinge:38	Hinge:39	Hinge:40	Hinge:41	Hinge:42	Hinge:43	Hinge:44	Hinge:45	Hinge:46	Hinge:47	Hinge:48	Hinge:49	Hinge:50	Hinge:51	Hinge:52	Hinge:53	Hinge:54	ASN #
	Hinge																																																	
MatureLinear #	221.9	221.10	221.11	221.12	221.13	221.14	221.15	221.16	221.17	221.18	221.19	221.20	221.21	221.22	221.23	221.24	221.25	221.26	221.27	221.28	221.29	221.30	221.31	221.32	221.33	221.34	221.35	221.36	221.37	221.38	221.39	221.40	221.41	221.42	221.43	221.44	221.45	221.46	221.47	221.48	221.49	221.50	221.51	221.52	221.53	221.54	221.55	221.56	221.57	221.58	MatureLinear #
3BNC117_HC	-	-	-	-	-	-	-	-	-	-	-	-	-	-	-	-	-	-	-	-	-	-	-	-	-	-	-	-	-	-	-	-	-	-	-	-	-	-	-	-	-	-	-	-	-	-	-	-	-	-	3BNC117_HC
MatureLinear #	230.9	230.10	230.11	230.12	230.13	230.14	230.15	230.16	230.17	230.18	230.19	230.20	230.21	230.22	230.23	230.24	230.25	230.26	230.27	230.28	230.29	230.30	230.31	230.32	230.33	230.34	230.35	230.36	230.37	230.38	230.39	230.40	230.41	230.42	230.43	230.44	230.45	230.46	230.47	230.48	230.49	230.50	230.51	230.52	230.53	230.54	230.55	230.56	230.57	230.58	MatureLinear #
PGT121_HC	-	-	-	-	-	-	-	-	-	-	-	-	-	-	-	-	-	-	-	-	-	-	-	-	-	-	-	-	-	-	-	-	-	-	-	-	-	-	-	-	-	-	-	-	-	-	-	-	-	-	PGT121_HC

ASN #	Region	MatureLinear # (3BNC117_HC)	3BNC117_HC	MatureLinear # (PGT121_HC)	PGT121_HC
Hinge:55	Hinge	221.59	-	230.59	-
Hinge:56		221.60	-	230.60	-
Hinge:57		221.61	-	230.61	-
Hinge:58		221.62	-	230.62	-
Hinge:59		221.63	-	230.63	-
Hinge:60		221.64	-	230.64	-
Hinge:61		221.65	-	230.65	-
Hinge:62		221.66	-	230.66	-
Hinge:63		221.67	-	230.67	-
Hinge:64		221.68	-	230.68	-
Hinge:65		221.69	-	230.69	-
Hinge:66		221.70	-	230.70	-
Hinge:67		221.71	-	230.71	-
Hinge:68		221.72	-	230.72	-
Hinge:69		221.73	-	230.73	-
Hinge:70		221.74	-	230.74	-
Hinge:71		221.75	-	230.75	-
Hinge:72		221.76	-	230.76	-
Hinge:73		221.77	-	230.77	-
Hinge:74		221.78	-	230.78	-
Hinge:75		221.79	-	230.79	-
Hinge:76		221.80	-	230.80	-
Hinge:77		221.81	-	230.81	-
Hinge:78		221.82	-	230.82	-
Hinge:79		221.83	-	230.83	-
Hinge:80		221.84	-	230.84	-
Hinge:81		221.85	-	230.85	-
Hinge:82		221.86	-	230.86	-
Hinge:83		221.87	-	230.87	-
Hinge:84		221.88	-	230.88	-
Hinge:85		221.89	-	230.89	-
Hinge:86		221.90	-	230.90	-
Hinge:87		221.91	-	230.91	-
Hinge:88		221.92	-	230.92	-
Hinge:89		221.93	-	230.93	-
Hinge:90		221.94	-	230.94	-
Hinge:91		221.95	-	230.95	-
Hinge:92		221.96	-	230.96	-
Hinge:93		221.97	-	230.97	-
Hinge:94		221.98	-	230.98	-
Hinge:95		221.99	-	230.99	-
Hinge:96		222	E	231	E
Hinge:97		223	P	232	P
Hinge:98		224	K	233	K
Hinge:99		225	S	234	S
Hinge:100		225.1	-	234.1	-
Hinge:101		225.2	-	234.2	-
Hinge:102		225.3	-	234.3	-
Hinge:103		225.4	-	234.4	-
Hinge:104		225.5	-	234.5	-

ASN #	Region	MatureLinear # (3BNC117_HC)	3BNC117_HC	MatureLinear # (PGT121_HC)	PGT121_HC
Hinge:105	Hinge	225.6	-	234.6	-
Hinge:106		225.7	-	234.7	-
Hinge:107		226	C	235	C
Hinge:108		227	D	236	D
Hinge:109		228	K	237	K
Hinge:110		229	T	238	T
Hinge:111		230	H	239	H
Hinge:112		231	T	240	T
Hinge:113		232	C	241	C
Hinge:114		233	P	242	P
Hinge:115		234	P	243	P
Hinge:116		235	C	244	C
Hinge:117		236	P	245	P
Hinge:118		237	A	246	A
Hinge:119		238	P	247	P
Hinge:120		239	E	248	E
Hinge:121		240	L	249	L
Hinge:122		241	L	250	L
Hinge:123		242	G	251	G
Fc-N:1	CH2	242.1	-	251.1	-
Fc-N:2		242.2	-	251.2	-
Fc-N:3		242.3	-	251.3	-
Fc-N:4		242.4	-	251.4	-
Fc-N:5		243	G	252	G
Fc-N:6		244	P	253	P
Fc-N:7		245	S	254	S
Fc-N:8		246	V	255	V
Fc-N:9		247	F	256	F
Fc-N:10		248	L	257	L
Fc-N:11		249	F	258	F
Fc-N:12		250	P	259	P
Fc-N:13		251	P	260	P
Fc-N:14		251.1	-	260.1	-
Fc-N:15		252	K	261	K
Fc-N:16		253	P	262	P
Fc-N:17		254	K	263	K
Fc-N:18		254.1	-	263.1	-
Fc-N:19		255	D	264	D
Fc-N:20		256	T	265	T
Fc-N:21		257	L	266	L
Fc-N:22		258	M	267	M
Fc-N:23		259	I	268	I
Fc-N:24		260	S	269	S
Fc-N:25		261	R	270	R
Fc-N:26		262	T	271	T
Fc-N:27		263	P	272	P
Fc-N:28		264	E	273	E
Fc-N:29		265	V	274	V
Fc-N:30		266	T	275	T
Fc-N:31		267	C	276	C

ASN #	Region	MatureLinear # (3BNC117_HC)	3BNC117_HC	MatureLinear # (PGT121_HC)	PGT121_HC
Fc-N:32	CH2	268	V	277	V
Fc-N:33		269	V	278	V
Fc-N:34		270	V	279	V
Fc-N:35		271	D	280	D
Fc-N:36		272	V	281	V
Fc-N:37		273	S	282	S
Fc-N:38		274	H	283	H
Fc-N:39		275	E	284	E
Fc-N:40		276	D	285	D
Fc-N:41		277	P	286	P
Fc-N:42		278	E	287	E
Fc-N:43		279	V	288	V
Fc-N:44		280	K	289	K
Fc-N:45		281	F	290	F
Fc-N:46		282	N	291	N
Fc-N:47		283	W	292	W
Fc-N:48		283.1	-	292.1	-
Fc-N:49		284	Y	293	Y
Fc-N:50		285	V	294	V
Fc-N:51		286	D	295	D
Fc-N:52		287	G	296	G
Fc-N:53		288	V	297	V
Fc-N:54		289	E	298	E
Fc-N:55		289.1	-	298.1	-
Fc-N:56		289.2	-	298.2	-
Fc-N:57		290	V	299	V
Fc-N:58		291	H	300	H
Fc-N:59		292	N	301	N
Fc-N:60		293	A	302	A
Fc-N:61		294	K	303	K
Fc-N:62		295	T	304	T
Fc-N:63		296	K	305	K
Fc-N:64		297	P	306	P
Fc-N:65		298	R	307	R
Fc-N:66		299	E	308	E
Fc-N:67		300	E	309	E
Fc-N:68		301	Q	310	Q
Fc-N:69		301.1	-	310.1	-
Fc-N:70		301.2	-	310.2	-
Fc-N:71		301.3	-	310.3	-
Fc-N:72		301.4	-	310.4	-
Fc-N:73		302	Y	311	Y
Fc-N:74		303	N	312	N
Fc-N:75		304	S	313	S
Fc-N:76		305	T	314	T
Fc-N:77		306	Y	315	Y
Fc-N:78		307	R	316	R
Fc-N:79		308	V	317	V
Fc-N:80		309	V	318	V
Fc-N:81		310	S	319	S

ASN #	Region	MatureLinear # (3BNC117_HC)	3BNC117_HC	MatureLinear # (PGT121_HC)	PGT121_HC
Fc-N:82	CH2	311	V	320	V
Fc-N:83		312	L	321	L
Fc-N:84		313	T	322	T
Fc-N:85		314	V	323	V
Fc-N:86		315	L	324	L
Fc-N:87		316	H	325	H
Fc-N:88		317	Q	326	Q
Fc-N:89		318	D	327	D
Fc-N:90		319	W	328	W
Fc-N:91		319.1	-	328.1	-
Fc-N:92		320	L	329	L
Fc-N:93		321	N	330	N
Fc-N:94		322	G	331	G
Fc-N:95		323	K	332	K
Fc-N:96		324	E	333	E
Fc-N:97		324.1	-	333.1	-
Fc-N:98		324.2	-	333.2	-
Fc-N:99		325	Y	334	Y
Fc-N:100		326	K	335	K
Fc-N:101		327	C	336	C
Fc-N:102		328	K	337	K
Fc-N:103		329	V	338	V
Fc-N:104		330	S	339	S
Fc-N:105		331	N	340	N
Fc-N:106		332	K	341	K
Fc-N:107		333	A	342	A
Fc-N:108		334	L	343	L
Fc-N:109		335	P	344	P
Fc-N:110		335.1	-	344.1	-
Fc-N:111		335.2	-	344.2	-
Fc-N:112		336	A	345	A
Fc-N:113		337	P	346	P
Fc-N:114		338	I	347	I
Fc-N:115		339	E	348	E
Fc-N:116		340	K	349	K
Fc-N:117		341	T	350	T
Fc-N:118		342	I	351	I
Fc-N:119		343	S	352	S
Fc-N:120		344	K	353	K
Fc-N:121		345	A	354	A
Fc-N:122		346	K	355	K
Fc-N:123		347	G	356	G
Fc-C:1	CH3	347.1	-	356.1	-
Fc-C:2		348	Q	357	Q
Fc-C:3		349	P	358	P
Fc-C:4		350	R	359	R
Fc-C:5		351	E	360	E
Fc-C:6		352	P	361	P
Fc-C:7		353	Q	362	Q
Fc-C:8		354	V	363	V

ASN #	Region	MatureLinear # (3BNC117_HC)	3BNC117_HC	MatureLinear # (PGT121_HC)	PGT121_HC
Fc-C:9	CH3	355	Y	364	Y
Fc-C:10		356	T	365	T
Fc-C:11		357	L	366	L
Fc-C:12		358	P	367	P
Fc-C:13		359	P	368	P
Fc-C:14		359.1	-	368.1	-
Fc-C:15		360	S	369	S
Fc-C:16		361	R	370	R
Fc-C:17		362	D	371	E
Fc-C:18		362.1	-	371.1	-
Fc-C:19		363	E	372	E
Fc-C:20		364	L	373	M
Fc-C:21		364.1	-	373.1	-
Fc-C:22		364.2	-	373.2	-
Fc-C:23		365	T	374	T
Fc-C:24		366	K	375	K
Fc-C:25		367	N	376	N
Fc-C:26		368	Q	377	Q
Fc-C:27		369	V	378	V
Fc-C:28		370	S	379	S
Fc-C:29		371	L	380	L
Fc-C:30		372	T	381	T
Fc-C:31		373	C	382	C
Fc-C:32		374	L	383	L
Fc-C:33		375	V	384	V
Fc-C:34		376	K	385	K
Fc-C:35		377	G	386	G
Fc-C:36		378	F	387	F
Fc-C:37		379	Y	388	Y
Fc-C:38		380	P	389	P
Fc-C:39		380.1	-	389.1	-
Fc-C:40		380.2	-	389.2	-
Fc-C:41		381	S	390	S
Fc-C:42		382	D	391	D
Fc-C:43		383	I	392	I
Fc-C:44		384	A	393	A
Fc-C:45		385	V	394	V
Fc-C:46		386	E	395	E
Fc-C:47		387	W	396	W
Fc-C:48		387.1	-	396.1	-
Fc-C:49		388	E	397	E
Fc-C:50		389	S	398	S
Fc-C:51		390	N	399	N
Fc-C:52		391	G	400	G
Fc-C:53		392	Q	401	Q
Fc-C:54		393	P	402	P
Fc-C:55		393.1	-	402.1	-
Fc-C:56		393.2	-	402.2	-
Fc-C:57		394	E	403	E
Fc-C:58		395	N	404	N

ASN #	Region	MatureLinear # (3BNC117_HC)	3BNC117_HC	MatureLinear # (PGT121_HC)	PGT121_HC
Fc-C:59	CH3	396	N	405	N
Fc-C:60		397	Y	406	Y
Fc-C:61		398	K	407	K
Fc-C:62		399	T	408	T
Fc-C:63		400	T	409	T
Fc-C:64		401	P	410	P
Fc-C:65		402	P	411	P
Fc-C:66		403	V	412	V
Fc-C:67		404	L	413	L
Fc-C:68		405	D	414	D
Fc-C:69		405.1	-	414.1	-
Fc-C:70		405.2	-	414.2	-
Fc-C:71		405.3	-	414.3	-
Fc-C:72		405.4	-	414.4	-
Fc-C:73		406	S	415	S
Fc-C:74		407	D	416	D
Fc-C:75		408	G	417	G
Fc-C:76		409	S	418	S
Fc-C:77		410	F	419	F
Fc-C:78		411	F	420	F
Fc-C:79		412	L	421	L
Fc-C:80		413	Y	422	Y
Fc-C:81		414	S	423	S
Fc-C:82		415	K	424	K
Fc-C:83		416	L	425	L
Fc-C:84		417	T	426	T
Fc-C:85		418	V	427	V
Fc-C:86		419	D	428	D
Fc-C:87		420	K	429	K
Fc-C:88		421	S	430	S
Fc-C:89		422	R	431	R
Fc-C:90		423	W	432	W
Fc-C:91		423.1	-	432.1	-
Fc-C:92		424	Q	433	Q
Fc-C:93		425	Q	434	Q
Fc-C:94		426	G	435	G
Fc-C:95		427	N	436	N
Fc-C:96		428	V	437	V
Fc-C:97		428.1	-	437.1	-
Fc-C:98		428.2	-	437.2	-
Fc-C:99		429	F	438	F
Fc-C:100		430	S	439	S
Fc-C:101		431	C	440	C
Fc-C:102		432	S	441	S
Fc-C:103		433	V	442	V
Fc-C:104		434	M	443	M
Fc-C:105		435	H	444	H
Fc-C:106		436	E	445	E
Fc-C:107		437	A	446	A
Fc-C:108		438	L	447	L

ASN #	Region	MatureLinear # (3BNC117_HC)	3BNC117_HC	MatureLinear # (PGT121_HC)	PGT121_HC
Fc-C:109	CH3	439	H	448	H
Fc-C:110		439.1	-	448.1	-
Fc-C:111		439.2	-	448.2	-
Fc-C:112		440	N	449	N
Fc-C:113		441	H	450	H
Fc-C:114		442	Y	451	Y
Fc-C:115		443	T	452	T
Fc-C:116		443.1	-	452.1	-
Fc-C:117		444	Q	453	Q
Fc-C:118		445	K	454	K
Fc-C:119		446	S	455	S
Fc-C:120		447	L	456	L
Fc-C:121		448	S	457	S
Fc-C:122		449	L	458	L
Fc-C:123		450	S	459	S
HCnst-Po:1		451	P	460	P
HCnst-Po:2		452	G	461	G
HCnst-Po:3		453	K	462	K
